# Construction of the super pan-genome for the genus *Actinidia* reveals structural variations linked to phenotypic diversity

**DOI:** 10.1093/hr/uhaf067

**Published:** 2025-03-03

**Authors:** Haolin Wu, Wenjie Yang, Guanyong Dong, Quanjun Hu, Dawei Li, Jianquan Liu

**Affiliations:** Key Laboratory of Bio-Resource and Eco-Environment of Ministry of Education, College of Life Sciences, Sichuan University, 1st Ring Road, Chengdu, 610065, China; Department of Urology, Urologic Surgery Center, Xinqiao Hospital, Third Military Medical University (Army Medical University), No. 184 Xinqiao Street, Chongqing, 400037, China; Key Laboratory of Bio-Resource and Eco-Environment of Ministry of Education, College of Life Sciences, Sichuan University, 1st Ring Road, Chengdu, 610065, China; Technology Innovation Service Center, No.110 Jiangnan Road, Cangxi, 628400, China; Key Laboratory of Bio-Resource and Eco-Environment of Ministry of Education, College of Life Sciences, Sichuan University, 1st Ring Road, Chengdu, 610065, China; Key Laboratory of Plant Germplasm Enhancement and Specialty Agriculture, Wuhan Botanical Garden, The Chinese Academy of Sciences, No.1 Lumo Road, Wuhan, 430074, China; Key Laboratory of Bio-Resource and Eco-Environment of Ministry of Education, College of Life Sciences, Sichuan University, 1st Ring Road, Chengdu, 610065, China; State Key Laboratory of Grassland AgroEcosystem, College of Ecology, Lanzhou University, No.222 South Tianshui Road, Lanzhou, 730000, China

## Abstract

Kiwifruits, belonging to the genus *Actinidia*, are acknowledged as one of the most successfully domesticated fruits in the twentieth century. Despite the rich wild resources and diverse phenotypes within this genus, insights into the genomic changes are still limited. Here, we conducted whole-genome sequencing on seven representative materials from highly diversified sections of *Actinidia*, leading to the assembly and annotation of 14 haplotype genomes with sizes spanning from 602.0 to 699.6 Mb. By compiling these haplotype genomes, we constructed a super pan-genome for the genus. We identified numerous structural variations (SVs, including variations in gene copy number) and highly diverged regions in these genomes. Notably, significant SV variability was observed within the intronic regions of the *MED25* and *TTG1* genes across different materials, suggesting their potential roles in influencing fruit size and trichome formation. Intriguingly, our findings indicated a high genetic divergence between two haplotype genomes, with one individual, tentatively named *Actinidia × leiocacarpae*, from sect. *Leiocacarpae*. This likely hybrid with a heterozygous genome exhibited notable genetic adaptations related to resistance against bacterial canker, particularly through the upregulation of the *RPM1* gene, which contains a specific SV, after infection by *Pseudomonas syringae* pv. *actinidiae*. In addition, we also discussed the interlineage hybridizations and taxonomic treatments of the genus *Actinidia*. Overall, the comprehensive pan-genome constructed here, along with our findings, lays a foundation for examining genetic compositions and markers, particularly those related to SVs, to facilitate hybrid breeding aimed at developing desired phenotypes in kiwifruits.

## Introduction

**Figure 1:**
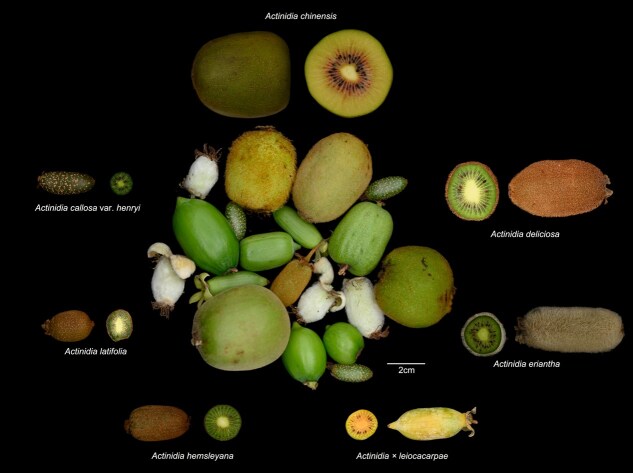
*Actinidia* taxa for pan-genome analysis and their fruit diversity. The central cluster displays various fruits of *Actinidia* taxa, while the surrounding images show the specific kiwifruit varieties used in this study, labeled with their corresponding tentative taxonomic names

Kiwifruit, a horticultural crop that has been domesticated recently, is renowned worldwide and often dubbed as ‘the king of fruits’ because of its remarkably high concentrations of vitamins, minerals, and other nutrients [1, 2]. The term ‘kiwifruits’ describes the fruits from different species belonging to the genus *Actinidia* (Actinidiaceae), known for their extensive genetic and morphological diversity [3] (Fig. 1). This genus contains around 54 ‘species’ and additional intraspecific taxa [2, 3], named primarily based on subjectively determined differences between morphological traits and further clustered into four sections: *Stellate*, *Strigosae*, *Maculatae*, and *Leiocacarpae*. Although all sampled taxa have the same basic chromosome number of *x* = 29, many currently defined ‘species’ encompass different ploidy entities, which show reproductive isolation (RI) and suggest that such ‘species’ may not comprise a single monophyletic and independently evolving lineage. Phylogenetic analyses based on sequence variations in mitochondrial, chloroplast, and nuclear genomes have revealed widespread topological inconsistencies, likely due to extensive hybridization and reticulate evolution among the existing ‘species’ of the genus [4–6]. Many ‘species’ may comprise multiple independently evolving lineages (IEL) with distinct RIs, including different ploidy levels, while some may be established based on hybrids produced from interlineage crossings that sometimes retain for a long time with many clonal offsprings. Such hybrids are not IELs that are required for the establishment of ‘species’, ‘subspecies’, even ‘variety’ according to the currently accepted taxonomic classification [7]. Therefore, the species delimitation and further material identification within *Actinidia* remain challenging and complicated before the final integrated taxonomic revision.

To date, more than 250 kiwifruit cultivars have been propagated clonally worldwide [6] and the majority originate from four tentatively named ‘taxa’: *A. chinensis* var. *chinensis*, *A. chinensis* var. *deliciosa*, *A. arguta,* and *A. eriantha* [8]. Many of these cultivars are susceptible by bacterial canker, resulting from the gram-negative bacterium *Pseudomonas syringae* pv. *actinidiae* (Psa). This condition frequently results in decreased yields and substantial economic losses across all principal kiwifruit-producing regions [9, 10]. Psa is currently assumed to comprise five biovars (biovars 1, 2, 3, 5, and 6) [11]. Psa biovar 3 (Psa3) is the most widespread and damaging strain, responsible for global pandemics over the past decade. It poses a significant threat to all commercial kiwifruit cultivars, with a particularly severe impact on the widely cultivated *A. chinensis* cv. ‘Hongyang’. Interestingly, some wild materials from sect. *Leiocacarpae*, including some ascribed to *A. polygama*, have been found to be resistant to Psa [12]. Examining the genomic changes in such materials could be beneficial for breeding new cultivars by transferring this elite trait.

Through the collection and cultivation of more than 1000 wild *Actinidia* materials across China in a common kiwifruit garden for over 20 years, we have identified a single wild female individual that is highly resistant to Psa (Fig. S1). This material is taxonomically similar to those of sect. *Leiocacarpae*, but with an uncertain ‘species’ ascription due to incomplete morphological traits and widespread species-delimitation issues, as mentioned previously [4–6]. We have provisionally assigned this female individual a hybrid name *A. × leiocacarpae* and used its clonal cutting for all experiments. We have assembled the *de novo* genome of this individual. Additionally, to enable comparison with other materials cultivated within the same garden, we performed whole-genome sequencing and analyses on six other representative cultivars or wild collections that are tentatively identified as six ‘species’ from the other three sections. Among them, *A. × leiocacarpae* (GZ) belongs to sect. *Leiocacarpae*, *A. callosa* var. *henryi* (JL) belongs to sect. *Maculatae*, *A. hemsleyana* (CY) belongs to sect. *Strigosae*, *A. latifolia* (KY), *A. eriantha* (MH), *A. deliciosa* (MW), and *A*. *chinensis* cv. ‘Hongyang’ (HY) belong to Sect. *Stellate* (Fig. 1). Voucher specimens of these seven female individuals have been collected and deposited, and their taxonomic names may be revised when the complete taxonomic review of the genus is completed according to the integrative species concepts and more practical criteria [7].

The seven materials selected encompass a broad spectrum of diversity within the *Actinidia* genus, exhibiting traits ranging from Psa resistance to variations in fruit size and trichome characteristics (Fig. 1). Our objective was to utilize these genomes to assemble a super-pangenome and to pinpoint the structural variations (SVs, with >50 bp intermaterial variations that also include gene losses and acquirements) that likely underlie these phenotypic diversities. Recent advances in pan-genomics have underscored the pivotal role of SVs in enhancing genetic diversity and facilitating adaptive processes across a range of species [13–15]. For example, the grapevine pan-genome revealed key SVs that underlie environmental adaptability and resistance to biotic stresses, providing valuable insights for breeding programs [16]. Similarly, in maize and wheat, pan-genomic analyses uncovered patterns of gene acquisition and loss and other SVs that are associated with traits such as stress tolerance and yield improvement [17, 18]. For fruit crops, pan-genomic studies in apple (*Malus*), jujube (*Ziziphus jujuba*), and apricot (*Prunus zhengheensis*) have identified SVs linked to key traits such as fruit size, flavor, sugar content, and environmental adaptations [19–21].

In our study, we applied pan-genomic approaches to the genus *Actinidia* to investigate its extensive genetic diversity and adaptability. It should be noted that a pan-genome of *A. chinensis* [22] and several genomes of diploid *Actinidia* taxa are already available, including *A. chinensis* [2, 3, 23–26], *A. eriantha* [8, 26–28], *A. deliciosa* [29], *A. latifolia* [24], *A. hemsleyana* [30], and *A. zhejiangensis* [30] (should be revised to be *A. × zhejiangensis* because it is F1 hybrid [7]). Additionally, a pan-genome encompassing 15 published *Actinidia* genomes has recently been reported [31]. However, we did not include these previously reported genomes in our pangenomic analyses. This decision was made to maintain consistency in all data and analytical parameters [32]. Our analysis exclusively relies on newly sequenced haplotype-resolved genomes to ensure methodological consistency and enable more accurate comparisons within the context of our study.

By integrating multispecies and haplotype-level genomic data from seven selected materials, we examined gene numbers and evolutionary rates for core and dispensable genes at the genus level. This analysis identified numerous SVs that are associated with phenotypic diversity, including traits such as fruit morphology and disease resistance. These findings provide valuable insights into the evolutionary mechanisms underlying these trait variations and offer useful resources for breeding programs focused on kiwifruit quality and environmental adaptability.

## Results

### Genome sequencing and assembly

To construct the super pan-genome of the genus *Actinidia*, seven diploid materials from four sections of the genus were selected for whole genome sequencing. For each taxon, one female individual was used. Leaf and flower specimens of each sequenced individual are shown in Fig. S1. The estimated genome sizes for the seven materials ranged from 586 to 647 Mb based on k-mer distribution analysis, and from 605 to 706 Mb according to flow cytometry (Figs S1–S3). We assembled each material by combining PacBio HiFi long reads sequencing and high-throughput chromosome conformation capture (Hi-C) technologies (Table S1). A total of seven primary genomes and 14 haplotype genomes were assembled, resulting in contig N50 sizes to be 8.4–22.5 Mb and overall assembly sizes to be 602.0–699.6 Mb (Table S2). In addition, about 90.0%–97.5% of genome sequences are successfully anchored to chromosomes (Table S2 and Fig. S4). In order not to conflict with the name of the primary genome, the haplotype genomes of each material were renamed (Table S2).

The assembled genomes showed high mapping rates for transcriptome data, with an average mapping rate of 91.6% (Table S3). Completeness analysis revealed that all 21 assemblies surpassed 99% according to BUSCO assessments (Fig. S5). Furthermore, LAI scores were higher than 16, indicating that the assemblies might be categorized as reference and even gold levels (Fig. S6). Collectively, these results demonstrate these genome sequences using HiFi sequencing technology are of high quality. Repetitive sequences were identified in each assembled genome, ranging from 38.7% to 47.3% of the genome size (239.5–331.1 Mb; Table S1). A significant correlation was observed between repeat sequence proportions and genome assembly sizes (*R* = 0.96, *P* = 4.549e-4 in primary genomes; *R* = 0.73, *P* = 2.9e-3 in haplotype genomes, Fig. S7). Among the repetitive elements, long-terminal repeat retrotransposons (LTR-RTs) were the most numerous, constituting 26.6% to 33.4% of the genomes (Table S4). A total of 3017–4936 intact LTR-RTs were identified across the seven *Actinidia* materials analyzed. These LTR-RTs were primarily classified into the Copia and Gypsy families, which accounted for 39.8% to 44.5% and 43.7% to 49.6% of the total intact LTR-RTs, respectively (Table S4). Sequence divergence between the LTR pairs revealed that most intact Gypsy and Copia elements expanded within the past one million years (Mya, Fig. S8), but the relative abundance of these two families varied across materials. In *A. callosa* var. *henryi*, Gypsy elements were slightly more abundant than Copia elements (2028 vs. 1917), suggesting a dominance of Gypsy activity. In contrast, both *A. × leiocacarpae* and *A*. *chinensis* cv. ‘Hongyang’ exhibited Copia-dominated patterns, with Copia elements significantly outnumbering Gypsy elements (*A. × leiocacarpae*: 1709 vs. 912; *A*. *chinensis* cv. ‘Hongyang’: 1747 vs. 996). These findings highlight lineage-specific differences in the transpositional dynamics of LTR-RT families across *Actinidia* materials.

**Figure 2:**
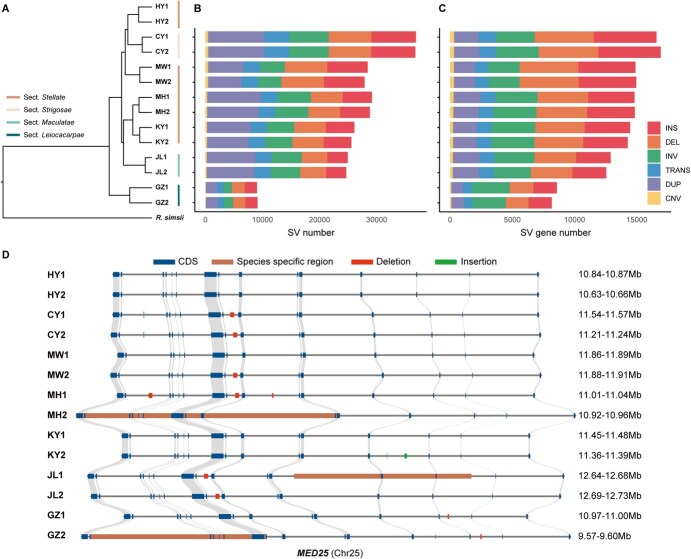
Phylogenetic relationships and SVs detected in assembled haplotype genomes. A Phylogenetic relationship among haplotype genomes. The four sections of *Actinidia* are shaded by different colors (*R. simsii* as outgroup); B SV number and C the number of genes containing SVs detected in 12 assembled haplotype genomes (use HY1 as the reference); D The landscape of variations of *MED25* gene in 14 assembled haplotype genomes. Syntenic regions, CDS regions, deletion regions, insertion region and species-specific region are indicated in different colors.

Gene annotation predicted 41 368 to 45 514 protein-encoding genes across the primary and haplotype genomes. The average coding sequence (CDS) length ranged from 1292 to 1353 bp, while the mean number of exons per gene was 5.7–6.3 (Table 1). Conserved core proteins were identified using BUSCO, with 1560–1592 (more than 96%) conserved core proteins detected across all assemblies (Table 1). In addition, 92.6–96.4% of the genes (39598–42 136) were clustered into 21 387–22 744 gene families (Fig. S9 and Table S5). Phylogenetic analysis based on 3067 single-copy orthologous genes revealed that the two haplotype genomes of *A. × leiocacarpae* were located at the earliest diverged position of the sampled *Actinidia* materials (Fig. 2A). Interestingly, the phylogenetic trees based on each chromosome displayed discrepancies with the overarching genome-wide phylogenetic tree (Fig. S10). *A. × leiocacarpae* has exceptionally higher heterozygosity (2.9%) than others. We identified five distinct categories of duplicate genes, including whole-genome duplication (WGD), tandem duplication (TD), proximal duplication (PD), transposed duplication (TRD), and dispersed duplication (DSD). Among these, 3281–4337 genes (7.8–9.5%) were classified as singletons, meaning they were only present in one genome (Fig. S11 and Tables S6 and S7). Genes derived from whole-genome duplication (WGD) events exhibited unique characteristics, including longer coding sequences (CDS), a higher number of CDS regions per gene, and lower *Ka*/*Ks* values compared to other categories of duplicate genes (Figs S12–S14).

**Table 1:** Genome annotations statistics for seven sequenced *Actinidia* materials.

**Materials**	**Accession**	**Assembled genome (Mb)**	**Repeat sequences (%)**	**Number of genes**	**Gene busco (%)**	**Annotated gene percentage (%)**
***A. callosa* var. *henryi***	**JL**	**699.6**	**45.3**	**45 514**	**98.3**	**97.2**
	**JL1**	**662.9**	**42.1**	**42 522**	**98.1**	**97.1**
	**JL2**	**656.8**	**44.2**	**41 520**	**97.9**	**97.1**
***A. chinensis* cv. ‘Hongyang’**	**HY**	**623.4**	**42.6**	**41 750**	**98.4**	**97.0**
	**HY1**	**610.4**	**41.6**	**41 550**	**98.6**	**97.2**
	**HY2**	**602.0**	**41.7**	**41 368**	**98.4**	**97.0**
***A. deliciosa***	**MW**	**630.1**	**43.0**	**41 993**	**98.4**	**97.1**
	**MW1**	**618.9**	**38.7**	**42 453**	**98.5**	**97.0**
	**MW2**	**610.9**	**39.3**	**42 290**	**98.7**	**97.1**
***A. eriantha***	**MH**	**653.4**	**45.6**	**42 280**	**97.8**	**96.9**
	**MH1**	**647.7**	**44.2**	**42 187**	**98.2**	**96.8**
	**MH2**	**630.4**	**44.1**	**41 675**	**97.8**	**96.9**
***A. hemsleyana***	**CY**	**674.5**	**45.2**	**42 211**	**96.7**	**97.3**
	**CY1**	**634.8**	**43.6**	**41 466**	**98.1**	**97.4**
	**CY2**	**634.8**	**43.1**	**41 649**	**98.6**	**97.4**
***A. latifolia***	**KY**	**699.6**	**47.3**	**42 440**	**96.7**	**97.0**
	**KY1**	**610.4**	**42.1**	**42 661**	**98.0**	**97.0**
	**KY2**	**650.0**	**41.8**	**42 215**	**97.9**	**97.0**
***A. × leiocacarpae***	**GZ**	**673.7**	**42.6**	**42 001**	**97.6**	**96.5**
	**GZ1**	**624.0**	**41.7**	**43 426**	**98.3**	**96.5**
	**GZ2**	**628.1**	**40.8**	**42 088**	**98.5**	**96.8**

### Super-pangenome of *Actinidia*

**Figure 3:**
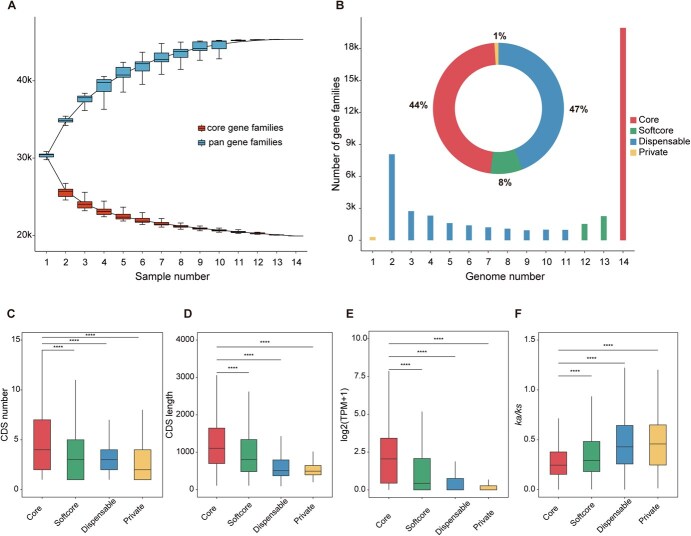
The super pan-genome of the genus *Actinidia*. A Number of core gene families and pan gene families for various combinations of haplotype genomes. B Proportion of core, softcore, dispensable, and private gene families. C CDS numbers, D CDS length, E leaf expressions and F selective constraints of core, softcore, dispensable and private genes in 14 haplotype genomes. In C-F, the statistical difference between groups was calculated using *t*-tests: ****P < 0.0001

We constructed super-pangenome of the *Actinidia* genus based on 14 haplotype genomes that were sampled, assembled, and annotated by the same processes. We annotated 45 349 gene families by clustering a total of 579 516 nonredundant genes from these genomes (Fig. 3A). Among these, 19 932 (44%) gene families shared by all 14 genomes were defined as core gene families; 3793 (8%) gene families were present in 12–13 genomes defined as softcore gene families; 21 320 (47%) gene families were present in 2–11 genomes defined as dispensable gene families; and 304 (1%) gene families were present in only one genome defined as private gene families (Fig. 3B). The proportion of dispensable gene families (47%) was the highest, indicating that there was a wide range of variation between different materials in the genus *Actinidia*. Besides, the proportion of core gene families (44%) was slightly lower than dispensable gene families, which was lower than that in rice (46%) [33], soybean (51%) [34], and pearl millet (46.6%–52.1%) [35], but higher than that in sorghum (36%) [36]. Softcore gene families and private gene families accounted for only 8% and 1%, respectively. In addition, there were 9554 genes that existed exclusively in one single genome (subsequently called singleton genes). In general, each genome contains 66.4%–68.1% of core genes, 10.5%–11.9% of softcore genes, 17.0%–20.3% of dispensable genes, and 0.1%–0.8% of private genes (Table S8). Two haplotype genomes of *A. × leiocacarpae* contained the most private genes and singleton genes.

Compared with the other three types of genes, core genes were found to be significantly longer in CDS length and with more CDS (Fig. 3C and D and Figs S15 and S16). Moreover, core genes showed a higher expression level and a lower *Ka/Ks*, suggesting that these gene families evolve at a slower rate and exhibit greater functional conservation (Fig. 3E and F and Figs S17 and S18). For dispensable genes, softcore genes and core genes, WGD genes represented the highest proportion, comprising over half of the total genes, but the content of WGD-derived genes changed greatly in the private genes (Fig. S19). In addition, in all genomes, WGD-derived genes accounted for more than 70% of the core genes, and the proportion of other three type genes gradually decreased, while the proportion of other five types of duplicated genes gradually increased (Fig. S19). We also explored the association between these four types of genes and the identified SV genes. Overall, the number of SV genes in the core genes was the smallest, and then the proportion of softcore genes, dispensable genes and private genes gradually increased (Fig. S20).

### SVs between and within each material

In addition to gene duplications, other SVs also significantly contribute to plant growth, development, and adaptation to abiotic stress [13–15, 19–21]. To further explore SVs between and with materials, we explored SVs mainly based on haplotype genomes. We utilized 13 haplotype genomes in comparison with a reference haplotype genome to detect syntenic as well as rearranged regions through the use of SyRI [37] (Table S9). Six categories of representative SVs larger than 50 bp were extracted, which comprised copy number variations (CNV), inversions (INV), duplications (DUP), translocations (TRANS), deletions (DEL), and insertions (INS). The total count of SVs ranged from 9053 to 36 886, with the length of individual SVs spanning from 50 bp to a few kilobytes or even up to the Mb scale. INVs and SVs with a length of 1–5 Kb were the most dominant type of variations (Fig. S21 and Table S9). Compared with genes without SVs (SV−), genes with SVs (SV+) had significantly higher gene expression levels. However, in most haplotype genomes, *Ka/Ks* of these genes did not show significant differences (Figs S22 and S23).

Due to the remotest phylogenetic relationship with the reference genome (*A. chinensis* cv*.* ‘Hongyang’), the lowest number of SVs (9053 and 9151), the lowest number of large fragment SVs (>5Kb) and the number of SV genes (7174 and 6683) were detected in the two sets of haplotype genomes of *A. × leiocacarpae* (GZ1 and GZ2) (Fig. 2A-C). We further examined the highly diverged region (HDR) due to SVs between two haplotype genomes of each material. A total of 46.1–161.0 Mb of HDR related to 4790–16 205 genes were identified between two haplotype genomes of each material (Table S10). Among them, the HDR (161.0 Mb) and HDR genes (16205) identified in *A. × leiocacarpae* were much higher than those in the other six materials (Fig. S24).

We finally examined whether the genes with the identified SVs between different materials are linked to the phenotypic diversity of seven materials. *TTG1* has been identified as a gene linked to trichome formation in tea and participated in the formation of cucumber fruit bloom trichomes in cucumber [38, 39]. The intron region of this gene is the HDR region because of the SVs between seven materials (Fig. S25). *MED25*/*PFT1* has been showed related to fruit size and may negatively regulate fruit development of kiwifruit [40]. Similarly, we also found diverse SVs of this gene between genomes of several materials (Fig. 2D). Besides, we detected an orthologous gene of resistance protein interaction factor *RIN13*, which could positively enhance the resistance function of *RPM1*. There was a 60 bp specific deletion in the CDS region of this gene in *A. × leiocacarpae* (Fig. S26)*.*

### Allele-specific expression analysis related to SVs

Allele-specific expression (ASE) may be pivotal for plant development and survival, driving evolutionary processes and contributing to phenotypic diversity [41]. To identify the genes that exhibited ASE during development in the fruit of *A. × leiocacarpae*, the two haplotype genomes were combined and used as the reference genome for ASE analysis. We gathered fruits at nine different development stages as follows: 40, 60, 80, and 85 days post-anthesis (DPA), followed by 1 to 5 days after harvesting (DAH, Fig. S27). The four developmental stages, occurring from 40 to 85 DPA, will be referred to as A-D stages, while the five maturity stages, spanning 1 to 5 DAH, will be labeled as E-I stages. Around 168.3Gb of raw data were generated, with an average of 94.8% of the reads mapping to the combined haplotype genome (Table S11). Alleles were further identified by collinear relationships between two haplotype genomes, with a total of 80.4% of the annotated genes (34 802 out of 43 426 genes) having two identified alleles. A total of 5133 genes with TPM > =2 and |log_2_FC| > = 2 were identified as significant ASE genes (Fig. S28).

According to whether the expression bias of genes was consistent in different fruit developmental stages, ASE genes could be further divided into consistent ASE genes and inconsistent ASE genes. A total of 2321 consistent ASE genes were identified. In the potential parental genome, 1020 consistent ASE genes were highly expressed in GZ-SG1, and 1301 consistent ASE genes were highly expressed in GZ-SG2 (Table S12). GO enrichment indicated that the highly expressed ASE genes in GZ-SG1 were mainly concentrated in ethanol metabolism (GO:0006066), unsaturated fatty acid metabolism (GO:0033559), 6-phosphoglucose metabolism (GO:0051156, Table S13); the highly expressed ASE genes in GZ-SG2 were mainly concentrated in negative regulation of protein dephosphorylation (GO:0035308), ketone biosynthetic process (GO:0042181), cellular lipid metabolic process (GO:0044255) and terpene metabolic process (GO:0042214, Table S14). The remaining 2812 ASE genes were inconsistent ASE genes, which mainly concentrated in flavonoid metabolic process (GO:0009812) and various stress-related pathways (Table S15). Among the 5142 ASE genes, 1315 genes (25.6%) contained SV, and 817 genes had SV in the upstream 2 Kb region, indicating that SV region was one of the possible causes of allele expression differences.

### Biotic and abiotic tolerance and related genes to SVs

Through a repetitive method, two types disease resistance genes were identified, namely the pattern-recognition receptor (RLK-LRR) and the nucleotide-binding site with leucine-rich repeat (NBS-LRR) genes, across seven different kiwifruit materials [42, 43]. A total of 115–360 NBS-LRR genes and 289–321 NBS-LRR genes were present (Fig. S29). The lowest number of above two types of disease-resistant genes in *A. chinensis* cv. ‘Hongyang’, which was in agreement with the phenotype of the reported highly susceptible (HS) [12]. As a contrast, two materials (*A. × leiocacarpae* and *A. eriantha*) showed high resistant (HR) phenotype after Psa infection contained relative greater disease-resistance genes (Fig. S29).

**Figure 4:**
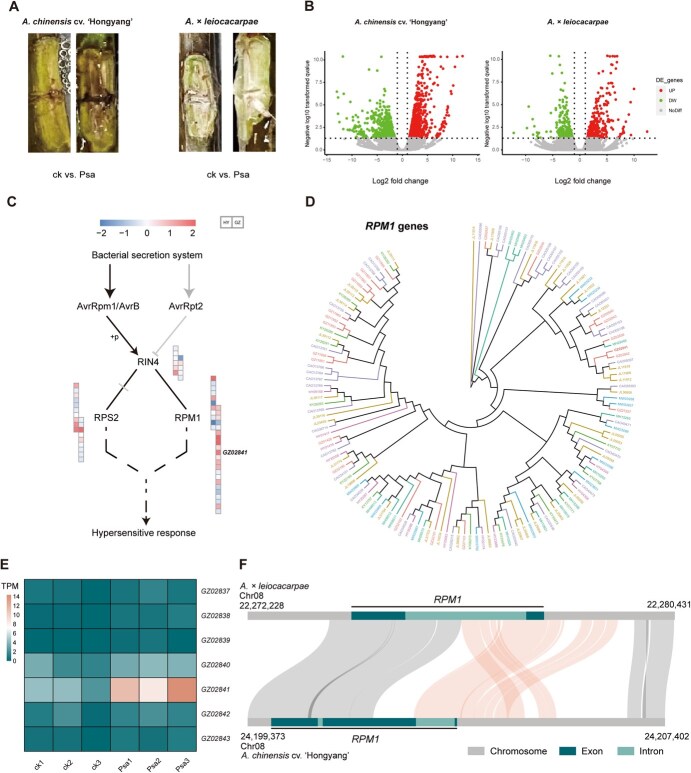
Disease-resistance genes and differential expression analysis of the *A. × leiocacarpae* genome. A The canes of *A. chinensis* cv. ‘Hongyang’ (left) and *A. × leiocacarpae* (right) were subjected to wound inoculation for 10 days, with Psa for treatment and sterile water for the control. B Volcano plots of the differential expression analysis. C The boxes aligned in a single row represent gene expression fold change for *A. chinensis* cv. ‘Hongyang’ on the left and *A. × leiocacarpae* on the right. D A phylogenetic tree displaying all candidate *RPM1* genes across seven genomes. E The expression of *RPM1* gene clusters on Chr08 in seven genomes. F Synteny between *A. × leiocacarpae* and *A. chinensis* cv. ‘Hongyang’ near *RPM1* gene, the translocation region was shown using orange line

To investigate the gene expression changes associated with the increased resistance to Psa in *A. × leiocacarpae*, RNA-seq data from the canes of *A. × leiocacarpae* and *A. chinensis* cv. ‘Hongyang’ were analyzed. The transcriptome of *A. × leiocacarpae*, which was highly resistant (HR) and *A. chinensis* cv. ‘Hongyang’, known for its high susceptibility (HS), were analyzed under conditions of Psa infection (designated as GZ-Psa and HY-Psa, respectively), as well as under a sterile water control treatment, labeled as GZ-ck for *A. × leiocacarpae* and HY-ck for *A. chinensis* cv. ‘Hongyang’*.* By the end of Psa treatment, *A. chinensis* cv. ‘Hongyang’ exhibited more severe stem necrosis (Fig. 4A). In total, 75.8 Gb clean data were produced from 11 RNA-seq samples, which were then aligned to their respective reference genomes, achieving mapping rates of 82.2% and 91.8%, respectively (Table S16). Analyses of differential gene expression were initially carried out through pairwise comparisons, specifically between GZ-Psa and GZ-ck, as well as HY-Psa and HY-ck. In the GZ-Psa vs. GZ-ck comparison, 546 differentially expressed genes (DEGs) were identified, comprising 303 that were up-regulated and 243 that were down-regulated. Conversely, when Ach-Psa was compared to Ach-ck, there were 1501 DEGs identified, with 842 being up-regulated and 659 down-regulated. This suggests that *A. × leiocacarpae* had fewer DEGs in response to Psa infection compared to *A. chinensis* cv. ‘Hongyang’ (Fig. 4B).

Additional analysis indicated that, among the orthologous genes between *A. chinensis* cv. ‘Hongyang’ and *A. × leiocacarpae*, 186 genes were distinctly up-regulated and 178 were uniquely down-regulated in the pairwise GZ-Psa vs. GZ-ck comparisons (Fig. S30). These specifically up-regulated genes were mainly involved in various metabolic processes, cell wall modification, defense, and response to various stimuli, while the specifically down-regulated genes were mainly concentrated in secondary metabolism and various stimulus–response pathways (Fig. S31). Further analysis showed that eight of the genes specifically up-regulated after Psa infection in *A. × leiocacarpae* were disease-resistant genes (Fig. S30). Among them, *GZ36249* (*BAM1*) gene could be in direct contact with the motility protein of tobacco flavivirus, suggesting that *BAM1* could be involved in the initial phases of viral transmission and the movement of viral proteins between cells [44, 45].

Further examination was conducted in *A. × leiocacarpae* for those 54 genes that were specifically up regulated after Psa infection, which did not have homologs in *A. chinensis* cv. ‘Hongyang’. GO enrichment revealed that these genes were predominantly associated with secondary metabolism and various stimulus–response pathways (Fig. S32). It is noteworthy that the *A. × leiocacarpae* owns one *RPM1* gene (*GZ02841*), whose expression level was significantly increased after Psa infection (log_2_FC = 1.64, FDR = 0.02). The *RPM1* gene was involved in an important effector-triggered immunity (ETI) resistance pathway and was resistant to *P. syringae* strains carrying the non-toxic genes *avrB* and *avrRpm1* (Fig. 4C), implying that this gene could have a crucial function after infection with Psa in *A. × leiocacarpae*. By comparing homologous genes with those in *A. thaliana*, we discovered all genes potentially participating in the ETI-related defense signaling pathway in both compared kiwifruit materials. We found that *A. × leiocacarpae* possesses a total of 22 *RPM1* genes, which was noticeably more than the 11 found in *A. chinensis* cv. ‘Hongyang’. We also focused on the copy number differences of the *RPM1* genes in other five materials. *A. callosa* var. *henryi* and *A. hemsleyana* had 35 genes, while there were only 14, 13, and 12 gene copies were found in *A. eriantha*, *A. latifolia* and *A. deliciosa*, respectively (Fig. 4D and Table S17). Among the 22 *RPM1* gene copies in *A. × leiocacarpae*, only 2 gene copies were singleton, and 11 gene copies of the remaining 20 copies (including *GZ02841*) were grouped in tandem repeat (TD) clusters (Table S17). *GZ02841* was in a tandem repeat gene cluster containing 7 *RPM1* gene copies on Chr08, while only 1 copy was contained in this collinear region in *A. chinensis* cv. ‘Hongyang’. In addition, the remaining five materials contained 12, 2, 4, 0, and 10 copies of the *RPM1* gene in same collinear region, respectively (Fig. S33). Among the 7 *RPM1* genes in *A. × leiocacarpae*, only *GZ02841* showed high expression after Psa infection (Fig. 4E). Therefore, we further examined the synteny of the genomic region (*GZ02841* and its orthologous gene) between *A. × leiocacarpae* and *A. chinensis* cv. ‘Hongyang’. The results revealed a significant translocation (~3Kb, Chr08: 22276186–22 279 120) within this region (Fig. 4F). Based on the structure annotation information, we discovered that this SV affects the last intron and exon of the *RPM1* gene, which may lead to an alteration in gene expression and function and subsequently impact disease resistance. Further analyses showed that this SV was not present in the other six materials.

### Comparative analysis of allele-specific expression and pan-genome DEGs in Psa infection

We also used combined haplotype genome of *A. × leiocacarpae* as references to explore the differential gene expression and identify genes that exhibit allele-specific expression (ASE) after Psa infection in *A. × leiocacarpae*. A total of 6 RNA-seq datasets (three biological replicates per stage) were calculated before and after infection with kiwifruit canker. Approximately, 41.6 Gb of raw data were produced, with an average mapping rate of 88.4% of the reads were aligned to the combined haplotype genome (Table S18). The average comparison rate of combined haplotype genome was improved by 6.2% (88.4% vs. 82.2%) compared with the reference primary genome of *A. × leiocacarpae* (Tables S14 and S16). We first identified 4432 DEGs in *A. × leiocacarpae* after the infection using combined haplotype genome as reference. Among these, 81.3% (3884) of the genes in *A. × leiocacarpae* belong to the pan-genome core gene families. Additionally, 11.5% (648) belong to the softcore gene family, 6.3% (299) belong to the dispensable gene family, and 0.1% (3) belong to the private gene family. This is similar to the situation observed in *A. chinensis* cv. ‘Hongyang’ (Fig. S34)*.* However, in terms of the overall count of differentially expressed genes, *A. × leiocacarpae* has fewer DEGs, aligning with previous results, which might be associated with its efficient disease resistance.

Furthermore, a total of 1729 genes with TPM > =2 and |log_2_FC| > = 2 were identified as significant ASE genes, which were further divided into 773 consistent ASE genes and 956 inconsistent ASE genes (Fig. S35 and Table S19). For the potential parental genome (GZ-SG1 and GZ-SG2), 397 consistent ASE genes showing high expression in GZ-SG1, and 376 consistent ASE genes showing high expression in GZ-SG2. Intriguingly, the expression of consistent ASE genes exhibits a clear expression bias. Concretely, the consistent ASE genes on 17 chromosomes were highly expressed in GZ-SG1, while the consistent ASE genes on the remaining 12 chromosomes were highly expressed in GZ-SG2 (Table S19). GO enrichment analysis revealed that the consistent ASE genes in GZ-SG1 were predominantly enriched in glutathione metabolism, triterpenoid biosynthesis and metabolism, cellular detoxification, regulation of jasmonate-mediated signaling pathway, and salicylic acid catabolism (Fig. S36 and Table S20). In addition, the consistent ASE genes in GZ-SG2 were mainly enriched in the regulatory pathways of various ion transmembrane transport (Fig. S36 and Table S21).

## Discussion

Kiwifruits are popular worldwide, but their domestication history are only a century, and their genetic background is relatively poor [8]. Most cultivated varieties originate from *A. chinensis* and *A. deliciosa*, with only a few from *A. arguta* and *A. eriantha*. The remaining genetic materials are mostly wild or semi-wild, limiting quality improvement.

Outbreaks of bacterial canker in kiwifruit have caused significant production losses, especially in highly commercialized varieties such as *A. chinensis* cv. ‘Hongyang’ and *A. deliciosa* cv. ‘Hayward’ [9, 10]. In contrast, some wild germplasm exhibits strong canker resistance. This study utilized HiFi sequencing and Hi-C technology to construct the primary and haplotype genomes of seven diploid materials from the genus *Actinidia*, including a highly resistant wild individual. We completed sequence annotation, gene structure prediction, and functional annotation for 21 genomes, constructing a genus-level super pangenome and identifying structural variants (SVs), which may contribute to phenotypic diversity, including disease resistance.

### Super-pangenome, SV, and phenotypic diversity

Given the substantial number of SVs across the seven *Actinidia* materials, we constructed a super pangenome based on the haplotype genomes. When the number of haplotype genomes reached approximately 12 to 13, the number of core gene families and pan gene families stabilized (Fig. 3A), indicating that these 14 genomes effectively capture genetic diversity across the whole *Actinidia* genus. Based on the super-pangenome, we identified a significant number of SVs between the two haplotype genomes within the same individual, which aligns with previous findings regarding *A. chinensis* cv. ‘Donghong’ [24]. SVs often impact the expression of nearby genes, potentially affecting evolutionary rates. It is intriguing that despite the increased expression levels of the SV-associated genes found in this research, the rate of molecular evolution showed no notable variations. One possible explanation is that these SVs may function as enhancers that boost gene expression without driving adaptive evolution. This is consistent with findings in other plants where SVs, particularly in noncoding regions, act as regulatory elements that enhance gene expression without significantly altering evolutionary trajectories [46, 47]. In *Actinidia*, this mechanism may underlie traits like disease resistance and fruit development, offering a key role for SVs in adaptive plasticity.

Our analysis suggests that SVs might contribute to the phenotypic diversity of kiwifruits through affecting expressions of the nearby genes. For instance, the *TTG1* gene, associated with plant epidermis formation, exhibited highly variable SVs across the seven sampled materials (Fig. S25). The sampled materials represent four sections of *Actinidia*, primarily classified based on epidermal morphological differences. Previous studies have suggested that this gene exhibited reduced or altered expression patterns between kiwifruit varieties with different epidermal characteristics [48]. These SVs may influence transcriptional regulation, potentially affecting key regions like promoters or enhancers, thus altering gene expression and affecting the phenotypic variations. For example, in *Arabidopsis thaliana*, SVs in the promoter regions of genes involved in trichome development have been shown to lead to noticeable differences in epidermal cell density [49]. A similar mechanism might be at play in *Actinidia*, where SVs could directly influence epidermal traits such as trichome density or glandular structures, contributing to the observed phenotypic diversity.

We also observed considerable variation in fruit size among different kiwifruit materials. Notably, the intron region of *MED25* displayed significant SVs between these materials (Fig. 2D). This gene has been studied for its association with fruit size in kiwifruit [40]. Additionally, variations in this gene have been shown to influence the size and maturation of different organs, including fruit and flora [50, 51]. The SVs in the intron region may disrupt splicing efficiency or affect intronic regulatory elements that control gene expression and then control the fruit size in kiwifruit materials. Further investigation with splice junction analysis could reveal how these SVs affect *MED25* expression and splicing. Similar findings have been observed in other crops, where intron-based SVs lead to altered gene splicing patterns, influencing traits like fruit size in *Solanaceae* [47, 52]. Alongside insertions and deletions, numerous base variants in different haplotype genomes may also influence fruit size variation among *Actinidia* species and materials.

In addition, we also showed that SVs may have contributed to canker resistance of kiwifruits. Overall, the extensive SVs identified among the seven materials, along with variations in gene copy numbers, likely contribute to the phenotypic diversity of kiwifruits, such as differences in fruit size and trichome developments. These SVs may also affect gene dosage, where variations in gene copy number could cause differences in expression levels, influencing developmental traits such as epidermal morphology and fruit size. However, all of these inferences need further confirmation by diverse methods.

### SVs may contribute to high canker resistance of *a*. × *leiocacarpae*

*A*. × *leiocacarpae* exhibited a highly resistant (HR) phenotype after Psa infection while *A. chinensis* cv. ‘Hongyang’ displayed a highly susceptible phenotype (HS, Fig. 4A). *A.* × *leiocacarpae* has the largest number of expanding gene families, including the disease-resistant *RPM1* genes, many of which resulted from tandem duplication. The observed expansion of *RPM1* gene copies in *A. × leiocacarpae* may enhance pathogen resistance through increased gene dosage, amplifying the production of *RPM1* protein and boosting effector-triggered immunity (ETI). Gene copy number variation (CNV) can also introduce functional redundancy, ensuring resistance even if some alleles are inactivated. Additionally, tandem duplications might enable subfunctionalization or neofunctionalization of *RPM1* paralogs, diversifying pathogen recognition capabilities. Our study also identified a specific deletion in the coding region of *RIN13* present only in *A.* × *leiocacarpae*, which may enhance the resistance function of *RPM1* (Fig. S26). In addition, we found that 54 genes that were uniquely and specifically up-regulated in *A. × leiocacarpae*, including the *RPM1* gene (GZ02841), which showed a significant increase after Psa infection (log2FC = 1.64, FDR = 0.02). Recent reports indicated that *RPM1* offered *A. thaliana* with resistance against strains of *P. syringae* [53, 54]. This up-regulation of *RPM1* and other related genes may indicate a strong activation of ETI, which is essential for resistance against Psa.

Moreover, our comparison between *A.* × *leiocacarpae* and *A. chinensis* cv. ‘Hongyang’ revealed a 3 kb translocation within the *RPM1* genomic region. This SV likely affects the last intron and exon of *RPM1*, potentially altering its function and contributing to the high resistance observed in *A.* × *leiocacarpae.* The presence of this SV in the *RPM1* region might facilitate its enhanced expression or function through a modified regulatory sequence, further strengthening the resistance against bacterial pathogens. Meanwhile, this SV was not present in the other six materials. Therefore, the significant SV variations in this region may help explain the differences in canker resistance among various kiwifruit materials. This SV aligns with findings in other plants where gene translocations have been associated with enhanced disease resistance. For example, in *A. thaliana*, translocations within resistance gene clusters have been shown to increase the diversity of effector recognition, thereby enhancing the overall immune response [55]. Similar observations were found in rice, where SVs in NLR gene loci not only strengthened disease resistance but also improved durability against evolving pathogen populations [56]. In *A. × leiocacarpae*, the identified translocation in the *RPM1* region may act through comparable mechanisms, altering regulatory sequences or introducing novel combinations of resistance gene alleles. These changes could expand the repertoire of pathogen recognition or improve the robustness of immune signaling, both of which are critical for combating Psa infections. The alteration of the *RPM1* region by SV could be a key factor in explaining the increased resistance in *A. × leiocacarpae* and should be studied further to confirm its role in pathogen recognition and response.

RIN4 (RPM1-interacting protein 4) is essential for the regulation of both effector-triggered immunity (ETI) and PAMP-triggered immunity (PTI) [54]. RPM1, along with the resistance protein RPS2, recognizes alterations in RIN4 triggered by three type III effectors from *P. syringae*: AvrRpt2, AvrRpm1 and AvrB. In vivo, AvrRpm1 and AvrB phosphorylate the T166 site of RIN4 to trigger RPM1 activation, whereas AvrRpt causes the breakdown of RIN4, leading to the activation of RPS2 [57–59]. Recent research on the genome o*f A. eriantha* also identified a similar ETI resistance pathway [27]. SVs that affect RIN4 or RPM1 expression can potentially modulate the immune response of plants by altering the effector recognition mechanism. Notably, HopF2 from *P. syringae* pv. *tomato* (Pst) DC3000 blocks the RIN4 degradation caused by AvrRpt2 [60]. However, both AvrRpt2 and AvrRpm1 are unable to be present together within the same bacterial strain, whether it is Pst or Psa [61]. Despite the presence of AvrRpm1 and AvrB in the *Psa3* genome, AvrRpt2 is lacking, indicating that RPM1 could be significant in providing disease resistance in the *Actinidia* genus [57]. In contrast, Pst JL1065 and Pst Q6LAD6 possess AvrRpt2 rather than AvrRpm1 and AvrB, while none of these genes are found in Pst DC3000 [57, 58, 62]. Additionally, two effector genes (AvrPtoB and AvrPto) from Pst DC3000 also contribute to the breakdown of RIN4 [63]. Coronatine is crucial for Pst DC3000 infection as it aids bacterial entry via stomata, whereas Psa3 is unable to produce this compound. These results indicate that Pst and Psa utilize distinct strategies to invade host plants.

### Inter-lineage hybridization, hybrid vigor and taxonomic treatment

**Figure 5:**
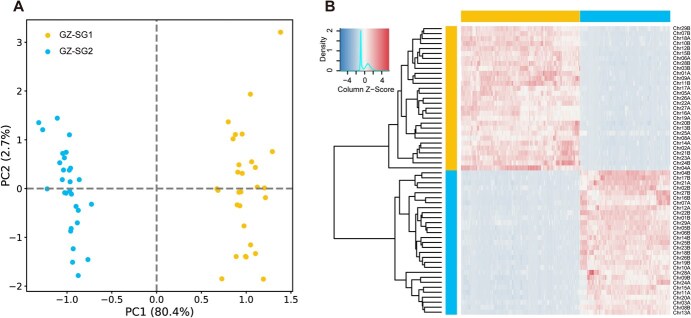
Phased parental haplotypes in *A. × leiocacarpae*. A PCA for Differential k-mers. B Heatmap and clustering of differential k-mers. The *x*-axis and *y*-axis represent differential k-mers and chromosomes, respectively.

Given the extensive hybridization between diverged *Actinidia* lineages [4–6], we further examined whether the seven sampled diploid materials had experienced past hybridizations between IELs. The heterozygosity of *A. × leiocacarpae* reached 2.9%, suggesting it likely originated from hybridization between two IELs. In addition, two haplotype genomes were well separated and assembled only for this material of seven sampled ones (Fig. 5 and Table S22) by SubPhaser [64]. The leaf and female flower characteristics of this individual resemble those of *A. polygama* or *A. kolomikta*. In contrast, a typical *A. polygama* individual collected from Wuhan Botanical Garden exhibited a heterozygosity of only 0.6%, indicating that the naturally homozygous *A. polygama* does exist as one IEL with the relatively pure homozygous genome. The higher heterozygosity observed in *A. × leiocacarpae* may have facilitated the maintenance of HDRs, which are enriched in adaptive genes, particularly those involved in disease resistance and abiotic stress tolerance. These HDRs are likely hotspots of recombination, allowing for the rapid emergence of beneficial alleles that enhance the fitness of hybrids in dynamic environments. The presence of HDRs in *A. × leiocacarpae* not only provides valuable insights into its evolutionary pressures and ecological adaptations but also poses significant challenges for SV detection. HDRs, characterized by their elevated sequence diversity and density of genetic elements, pose challenges for conventional SV detection methods. Advanced computational algorithms capable of refining the classification of HDRs into specific SV categories, such as insertions, deletions, or inversions, will be crucial for future studies. These approaches would allow for a more precise understanding of the genomic contributions of HDRs to phenotypic variations.

Meanwhile, the hybrids between distinct IELs of kiwifruits generally show high heterozygosity. For example, the sequenced genome of *A. zhejiangensis* was identified as an interspecific hybrid between *A. eriantha* and *A. hemsleyana,* displaying an exceptionally high heterozygosity of 7% [30]. In fact, *A. zhejiangensis* should be classified and named as an F1 hybrid, *A.* × *zhejiangensis* [7], rather than a distinct species as it is currently treated [30]. However, the diploid species with homoploid hybrid origin, should evolve from fertile offspring of a common ancestor over many generations [65–67]. In this context, all descendant individuals share a common genetic pool, resulting in significant gene flow across populations and individuals [7]. This genetic pool differentiates distinctly from other IELs (or true species) [7] although hybridizations between them produce the heterozygous offspring. For *A. × leiocacarpae*, the combination of two diverged haplotypes may also lead to hybrid vigor, enhancing both disease resistance and ecological adaptability through increased allelic diversity. The distinct expression patterns of alleles from its two haplotypes during fruit development and Psa infection (Tables S12 and S19) provide further evidence of heterosis, where hybrid-specific gene regulation could drive improved performance compared to either parental lineage. However, further examination of the extensive materials across the total distributions of sect. *Leiocacarpae* is badly needed to determine the hybrid origin and likely parents of *A. × leiocacarpae*.

Furthermore, the sampled hybrid here, *A*. × *leiocacarpae* likely originated from hybridization between two IELs within sect. *Leiocacarpae*, independent of the other three sections for the following reasons. First, phylogenetic analyses of six other materials representing these sections indicated that the two haplotype genomes of *A.* × *leiocacarpae* consistently clustered into a monophyletic group. Regrettably, we failed to include more samples from other IELs within sect. *Leiocacarpae,* making it challenging to identify the exact two evolutionary lineages or species from which this hybrid arose. The lower heterozygosity of *A.* × *leiocacarpae* compared to the hybrid *A.* × *zhejiangensis* suggests it may have originated from more recently diverged IELs within this section. Future research should involve sequencing the genomes of all diploid materials within sect. *Leiocacarpae* to better identify the likely parental IELs for this hybrid that was unintentionally collected in the wild. Additionally, these findings underscore the importance of preparing and preserving voucher specimens for all research materials, particularly those involved in genomic studies. The goals of such studies do not directly relate to taxonomic treatments, and therefore misnaming does not affect any related results and conclusions [7]. However, the voucher specimens are critical for future taxonomic revisions of all used materials in the past research and naming corrections according to the ‘Rules of Taxonomic Nomenclature’ and type specimens linked to its taxonomic latin names. *Actinidia* is a dioecious genus, and various species within the genus could hybridize to produce viable seeds, indicating F1 hybrids or introgressed individuals from IELs may widely exist among wild kiwifruits [6]. Utilizing integrative methods based on integrative species concept is essential [7] for identifying IELs to accurately delimit species and hybrids within wild kiwifruits. Such integrative studies not only enhance taxonomic treatments of the genus but also contribute greatly to cultivar breeding, as the wild hybrids between true species (IELs) can be directly utilized for commercial cultivation.

## Materials and methods

### Sample collection

The seven sequenced materials of kiwifruit were derived from a germplasm resource nursery in Shifang, Sichuan Province, China (31°13′46″N, 104°0′53″E). These materials are cultivated varieties and wild materials collected for crossbreeding, screening, and preservation of excellent germplasm for disease resistance. The wild materials in the field were collected randomly without strict classification and identification. Among the preserved materials, it was found that a wild material of *Actinidia* genus collected from Longmen Mountain was particularly resistant to kiwifruit canker disease, almost without any infection. This material was a female plant and belongs to sect. *Leiocacarpae*. Besides, the leaf and flower morphology of this material are more like *A. kolomikta*, but also similar to *A. polygama* (Fig. S1). Due to its strong ability to resist kiwifruit canker disease, more clonal offspring have been obtained through cutting, which was used as the rootstock of various cultivars of kiwifruit, and new varieties are being declared. Because hybridization between different ‘morphotaxonomic species’ of the whole *Actinidia* genus is extensive, species boundaries are still unclear. However, in the study results and description, there must be a name to describe, so we tentatively named this material as *Actinidia* × *leiocacarpae*. When studying fruit development of this only especial female individual, we had to use the male flowers of *A. polygama* to pollinate it. According to the leaf type and flower characteristics, the remaining six diploid materials were preliminarily identified as *A. callosa* var. *Henryi*, *A. chinensis* cv. ‘Hongyang’, *A. deliciosa*, *A. eriantha*, *A. hemsleyana*, and *A. latifolia*. Branches of all seven materials, including leaves, flowers and fruits (for *A.* × *leiocacarpae*, it is naturally pollinated by unknown pollens, probably from the other *A. kolomikta* or *A. polygama* male individuals) were collected from different parts of the flowering stage as voucher specimens (SCU-Art-Genome-01 to SCU-Art-Genome-07) and kept in herbarium of Sichuan University.

### HiFi sequencing and genome assembly

The fresh young leaves from above seven female materials were used for extracting high-quality genomic DNA with a DNAsecure Plant Kit (Tianjian Biotechnology Co., Ltd., Beijing, China). The HiFi libraries were built and then loaded onto the PacBio Sequel II/Sequel IIe platform by diffusion loading method for sequencing. Low-quality polymerase reads with sequencing adapters, length < 50 bp and quality score < 0.8 were disposed. The above subreads were processed using SMRTLink software to generate the final HiFi reads. Finally, we used Hifiasm [68] software to assemble the genome. For the same individual, this study separately assembled a set of primary genomes and two sets of haplotype-resolved genomes.

### Estimation of heterozygosity and genome size

Before assembling the genomes, we conducted a k-mer analysis to survey heterozygosity and estimate genome size. We first used corresponding HiFi reads to estimate the heterozygosity level with GenomeScope2 [69] k-mer spectroscopy analysis. We also performed k-mer analysis by GenomeScope2 [69] and KMC [70] software to estimate the genome size of different kiwifruit materials using the following formula:


\begin{align*} G=\frac{N\times \left(L-K+1\right)}{F} \end{align*}

in which *G*, *N*, *L*, *K*, *F* mean genome size, total reads number, read length, length of k-mer, and peak of k-mer frequency, respectively. To further estimate the genome sizes, we collected fresh tender leaves from the sequenced individual and analyzed them by flow cytometry (FCM) using *Populus alba* [71] as a standard.

### Chromosome-scale assembly using hi-C

For the Hi-C experiment, around 2 g of fresh young leaves from the sequenced kiwifruit individual were finely ground under liquid nitrogen. Then, we prepared the sequencing library on the Illumina NovaSeq 6000 platform. Using Juicer [72] and 3D-DNA [73], we assembled the contigs into pseudomolecules using the Hi-C reads. For obviously misordered or misoriented sequences, Juicebox [74] was used to manually adjust the final assembly result based on the interaction heat map. In order to facilitate subsequent comparative analysis and SV analysis, the chromosome identification and orientation were manually adjusted based on *A. deliciosa* using Minimap2 [75].

We employed three different methods to assess the quality of the genome assemblies. We first used BUSCO [76] with ‘embryophyta_odb10’ database and default parameters to evaluate the integrity of the assembled genome. Second, we assessed the mapping rate by aligning RNA-seq reads to the genome using HISAT2 [77]. Finally, the assembly continuity of each genome was assessed using LAI scores.

### Identification of repetitive sequences

In this study, we predicted the repeated sequences of various kiwifruit materials using two approaches: *de novo* search and homology alignment. First, RepeatMasker [78] with Repbase [79] (v.16.10) was applied to identify repeats in the genome assemblies. Next, RepeatModeler [80] was used to construct *de novo* transposable elements (TEs) libraries. Finally, by combining the above results, the final annotation set of repeated sequences was obtained by integrating the overlapping TE and removing the results of low scores and was used for subsequent gene prediction analysis.

We further identified candidate LTR-RTs using LTRharvest [81] and LTR_Finder (v1.02) [82]. Finally, we used the LTR_retriever [83] pipeline to integrate the results and eliminate false positives, enabling the identification of intact LTR-RTs. The insertion time of LTRs was then estimated using *T = K/2r*, where *K* denotes the divergence rate and *r* represents the base mutation rate, set at 3.39 × 10^−9^ for kiwifruit [2]. Besides, TEsorter [84] was used to classify LTR class transposons in kiwifruit genomes more accurately, so as to explore the inter-specific diversity of TE.

### Gene prediction and functional annotation

To predict protein-coding genes, we applied GETA (https://github.com/chenlianfu/geta), an automated genome-wide annotation tool that integrates transcriptome-based, homology-based, and *ab initio* evidence. Using the GETA pipeline, we directly aligned the RNA-Seq data generated in this study to the assembled genomes to provide transcript information. For homology-based prediction, protein sequences from *A. chinensis* cv. ‘Hongyang’, *A.* × *leiocacarpae*, *A. eriantha* and *R. simsii* were obtained from NCBI, Phytozome and KGD [85]. These sequences were then mapped to the corresponding genome using TBLASTN [86] with an E-value threshold of 1 × 10^−5^. According to the results of alignment, we further annotated the gene models using Genewise [87]. In the *ab initio* prediction step, Augustus [88] was executed on the masked genome, employing self-trained model parameters to identify candidate protein-coding genes. Predictions from all methods were then automatically merged by GETA to generate a consensus gene set.

Functional annotation of protein-coding genes was conducted using BLASTP [86] (selecting the best hit with E < 1e−5 and a maximum of 20 target alignments) based on the Swiss-Prot and TrEMBL databases [89]. Additionally, InterPro [90] was employed to identify conserved motifs and structural domains. Proteins from different kiwifruit genomes were submitted to EggNOG v5.0 database [91] for functional annotation. Furthermore, the GO annotation results of genes were finally obtained by integrating the GO results identified by the above four methods.

### Synteny and whole-genome duplication

Here, using *A. chinensis* cv. ‘Hongyang’ as the reference genome, collinear blocks (parameter: -icl) between two materials were determined based on WGDI [92], in which the collinear block was defined as containing at least ten collinear gene pairs. WGDI was also applied to obtain visual synteny information between different genomes (parameter: -d), as well as inter-and intra-material *Ka* and *Ks* calculations (parameter: -ks). Ks peaks were further corrected using the (−kf) command in this software. We further calculated the differentiation time corresponding to each *Ks* peak. In addition, based on WGDI software, the collinearity relationship between two haplotype genomes and the calculation of *Ka* and *Ks* between haplotypes in the same individual were further determined.

### Duplicate gene identification and positive selection

We used DupGen_finder [93] with default settings to classify gene duplication modes in each primary genome. The different duplicate genes could be divided into five types: whole-genome duplication (WGD), tandem duplication (TD), proximal duplication (PD), transposed duplication (TRD), and dispersed duplication (DSD). In this classification, PD genes are defined as duplicates with fewer than 10 gene intervals on the same chromosome, while TRD genes represent transposable duplicates. DSD genes include all other duplicates not classified within the four primary types. We used *R. simsii* as outgroup and all duplicated gene pairs in different kiwifruit materials were further inferred by a self-BLASTP and BLASTp search against *R. simsii*. In addition, to eliminate the potential impact of redundant duplicate genes between different types on subsequent analysis, this study divided all duplicate gene pairs into different duplicate gene types based on the GenDup_finder-unique command provided by DupGen_finder software and assigned them to a unique pattern. The classification priority for duplicated genes was assigned as follows: WGD > TD > PD > TRD > DSD. Subsequently, *Ka*/*Ks* ratios for each type of duplicated gene pair in each genome were estimated using WGDI [92]. For haplotype genomes, duplicate genes were classified using the same method.

To investigate signatures of positive selection, we identified one-to-one orthologous gene sets across seven *Actinidia* species and the *R. simsii* genome using OrthoFinder [94]. Protein sequences were aligned with MAFFT [95], and codon alignments were subsequently generated through PAL2NAL [96]. Positive selection analysis was then conducted using the branch-site model in Codeml from the PAML package [97]. Initial identification of genes under positive selection in *A. × leiocacarpae* was performed using Bayes Empirical Bayes (BEB) analysis. Following this, we applied a chi-square test from the PAML package, setting a significant threshold of *P* < 0.05.

### SV analysis

For SV analysis between materials, we first conducted pairwise genome alignments across each of the seven primary genomes (used *A. chinensis* cv. ‘Hongyang’ as reference genome) and fourteen haplotype genomes (used HY1 as reference genome) by minimap2 [75] software with default parameters. Next, we applied the SyRI pipeline [37] with default settings to identify and visualize structural variants (SVs). For detailed analysis, six SV types over 50 bp in length were selected, encompassing insertions (INS), deletions (DEL), inversions (INV), translocations (TRANS), duplications (DUP), and copy number variations (CNV). The ‘coverage’ command of BEDTools [98] software was used to screen the SV genes of the corresponding types. We further compared *Ka*/*Ks* and expression levels between genes with SV (SV+) and genes without SV (SV−).

For intra-material SV analysis, we firstly used minimap2 [75] software with default parameters to perform pairwise genome alignments within each materials (used haplotype genome 1 in corresponding material as reference genome). The rest of the analysis was performed as described in inter-material SV analysis.

### Construction the super-pangenome of *Actinidia* genus

For gene family clustering, OrthoFinder [94] was applied across 14 haplotype genomes derived from 7 diploid kiwifruit samples. To enhance analytical accuracy, genes containing premature stop codons or open reading frames (ORFs) shorter than 50 amino acids were excluded, and only the longest transcript from each gene was retained for clustering. Based on gene family distribution across the 14 genomes, the gene families were categorized into four classes: core, softcore, dispensable, and private. Core gene families were those shared by all 14 genomes, while softcore gene families appeared in 12–13 genomes (over 80%). Dispensable gene families were present in 2–11 genomes, and private gene families were found in only one genome. Additionally, we compared CDS length, CDS count, and expression levels across these pan-gene types to assess differences, the statistical difference between groups was calculated using *t*-tests.

### Haplotype genome origin and ASE analysis

SubPhaser [64] was used to further split the two sets of haplotype genomes of seven kiwifruit materials into two sets of parental genomes based on specific k-mer. For the two sets of parent genomes, the LTR classification and LTR insertion time were analyzed. For *A. × leiocacarpae* specifically, SubPhaser was used to phase its two haplotype genomes (GZ-SG1 and GZ-SG2) into parental subgenomes. This process involved counting k-mers using Jellyfish [99] clustering differential k-mers, defined as k-mers showing statistically significant differences in abundance between the two haplotypes, into subgenomes through a K-Means algorithm. Principal component analysis (PCA) and hierarchical clustering were performed to validate the phasing, and clear separation between GZ-SG1 and GZ-SG2 was observed. Furthermore, the distribution of differential k-mers across chromosomes was analyzed using the module for subgenome-specific k-mer enrichment provided by SubPhaser.

To identify genes displaying allele-specific expression (ASE) in *A. × leiocacarpae*, we analyzed RNA-seq data under two conditions: during fruit development and before and after Psa infection. For fruit development, expression levels were calculated from 27 RNA-seq datasets covering 9 developmental stages, with 3 biological replicates per stage. For the Psa infection analysis, 6 RNA-seq datasets were used, including 3 replicates each for the pre- and post-infection conditions. In order to avoid the possible dominant error when two haplotype genomes were used separately to calculate the expression level, a calculation strategy similar to Han [24] was used. First, the two haplotype genomes of *A.* × *leiocacarpae* were merged into a combined haplotype genome as the reference genome. After that, both RNA-seq datasets were aligned to this reference genome using HISAT2 [77] with default parameters, retaining only uniquely mapped reads for further analysis. We used the Stringtie [100] software package to calculate gene expression levels using the transcripts per million (TPM) method. Finally, JCVI [101] was employed to identify orthologous genes between the two haplotype genomes of *A. × leiocacarpae*, which were subsequently used to determine the fold change (FC) in gene expression levels. Any of the homologous genes with TPM > =2 and |log_2_FC| > = 2 were identified as significant ASE genes. ASE genes could be further divided into consistent ASE genes and inconsistent ASE genes according to their expression bias in different tissues, different developmental stages, or different stress treatments. Among them, consistent ASE genes are genes that are completely biased to be highly expressed in GZ1 or GZ2 in all transcriptomes, while inconsistent ASE genes are biased to be highly expressed in GZ1 in one transcriptome and lowly expressed in other transcriptomes.

### Identification and characterization of disease-resistance genes

To identify disease-resistance genes, we focused on two major types: nucleotide-binding site leucine-rich repeat (NBS-LRR) and pattern-recognition receptor RLK-LRR genes across seven *Actinidia* genomes. For NBS-encoding genes, we applied a widely used approach in plant studies [42, 43]. First, HMMER (v3.1b1) [102] was used with default parameters to extract protein sequences, screening them against the NB-ARC family hidden Markov model (HMM) profile (PF00931). High-confidence NBS sequences (E-value ≤1e-6) from these initial results were then aligned using MAFFT [95] and used to build specific NBS HMMs with ‘hmmbuild’ module. The refined NBS models were employed in a secondary search to finalize the NBS gene set (E-value ≤1e-5) in each genome.

To further characterize domain composition within NBS-encoding genes, we retrieved TIR (PF01582) and 11 LRR domain HMMs from the PFAM database (http://pfam.sanger.ac.uk/). These were then used to search against the finalized NBS-encoding protein set using HMMER (E-value ≤1e-2). The presence of TIR and LRR domains was validated using MEME (for motif identification) and the NCBI Conserved Domain Database (CDD).

For RLK-LRR genes, we again used HMMER (v3.1b1) [102] with default parameters, scanning predicted protein set of each genome against the RLK family HMM (PF00069) from PFAM. Candidate RLK proteins were further screened using the LRR HMM profiles to confirm their domain structure. This method provided a comprehensive set of NBS-LRR and RLK-LRR resistance genes across the seven kiwifruit genomes.

### Gene expression analysis in response to Psa infection

To investigate gene expression changes in response to Psa infection, we used a strain originally isolated from a kiwifruit canker area in Chengdu, Sichuan Province, identified as belonging to the Psa 3 group via PCR. The strain was activated on LB solid medium at 25°C for 36 h and then transferred to LB liquid medium, where it was cultured at 25°C and 250 rpm for 14 h. The bacterial suspension was diluted to 1 × 10^8^ CFU/mL in sterile water for inoculation. Healthy one-year-old canes of *A. × leiocacarpae* and *A. chinensis* cv. ‘Hongyang’ were prepared by sealing both ends with paraffin to prevent dehydration. Following surface sterilization with 70% alcohol, a 10 μl aliquot of Psa suspension was applied to wounds made in the vascular cambium; sterile water inoculation served as a control. Canes were incubated at 20°C with a 12-h light/dark cycle, and lesion lengths were recorded 10 days post-inoculation, with three biological replicates per sample.

To analyze gene expression responses, RNA-seq data from Psa-infected and control canes were mapped to the respective genomes using HISAT2 [77]. Gene expression levels were quantified with Stringtie [100] using the TPM method. OrthoFinder [94] was then used to identify orthologous genes between *A. × leiocacarpae* and *A. chinensis* cv. ‘Hongyang’, enabling fold change (FC) calculation in gene expression levels.

## Supplementary Material

Web_Material_uhaf067

## Data Availability

All raw sequence data and genome assembly data have been deposited in National Genomics Data Center, Beijing Institute of Genomics, Chinese Academy of Sciences and China National Center for Bioinformation, under accession number PRJCA031175.
