# Supplementary material for: Construction of the super pan-genome for the genus Actinidia reveals structural variations linked to phenotypic diversity
Source: Hortic Res. 2025 Mar 3;12(6):uhaf067. doi: 10.1093/hr/uhaf067 (PMC12038230; doi:10.1093/hr/uhaf067)
Supplement: Web_Material_uhaf067 [file web_material_uhaf067.zip › S_Information.v0217.docx]

**Supplementary Information of**

Construction of the super pan-genome for the genus *Actinidia* reveals structural variations linked to phenotypic diversity

Haolin Wu#^1,2^, Wenjie Yang#^1^, Guanyong Dong^3^, Quanjun Hu*^1^, Dawei Li*^4^, Jianquan Liu*^1,5^

^1^Key Laboratory of Bio-Resource and Eco-Environment of Ministry of Education, College of Life Sciences, Sichuan University, Chengdu 610065, China

^2^Department of Urology, Urologic Surgery Center, Xinqiao Hospital, Third Military Medical University (Army Medical University), Chongqing 400037, China

^3^Technology Innovation Service Center, Cangxi, Sichuan 628400, China

^4^Key Laboratory of Plant Germplasm Enhancement and Specialty Agriculture, Wuhan Botanical Garden, The Chinese Academy of Sciences, Wuhan, Hubei 430074, China

^5^State Key Laboratory of Grassland AgroEcosystem, College of Ecology, Lanzhou University, Lanzhou, China

#These authors contributed equally to this article.

*Corresponding authors: liujq@nwipb.ac.cn; lidawei@wbgcas.cn; huquanjun@scu.edu.cn

Haolin Wu: whl23@tmmu.edu.cn

Wenjie Yang: yangwj@stu.scu.edu.cn

Guanyong Dong: 465743419@qq.com

Quanjun Hu: huquanjun@scu.edu.cn

Dawei Li: lidawei@wbgcas.cn

Jianquan Liu: liujq@nwipb.ac.cn


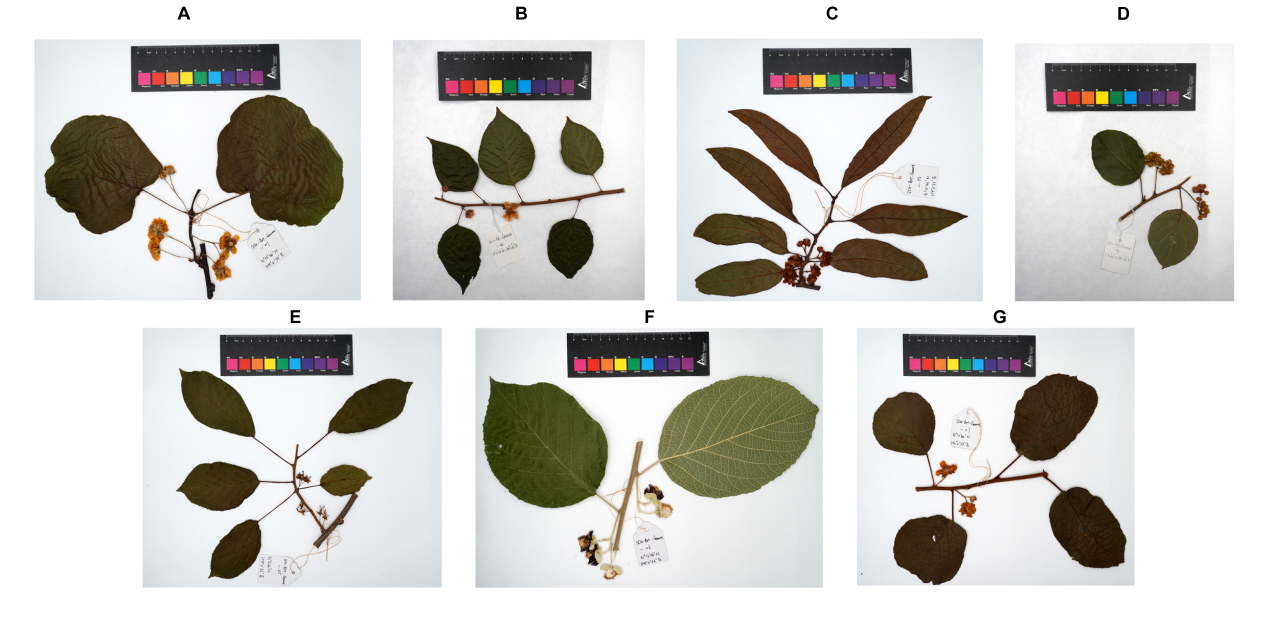


**Supplementary Figure 1.** Voucher specimens of 7 *Actinidia* materials used for genome sequencing in this study. A *A. chinensis* cv. ‘Hongyang’, B *A. × leiocacarpae*, C *A. hemsleyana*, D *A. latifolia*, E *A. callosa* var. *henryi*, F *A. eriantha* and G *A. deliciosa*. Three parts of each individual were collected for making voucher specimens in order to show the taxonomic characters of each individual. Here only one specimen was selected for each individual, showing the major taxonomic characters for such one individual, primarily focusing on the flowers and mature leaves.


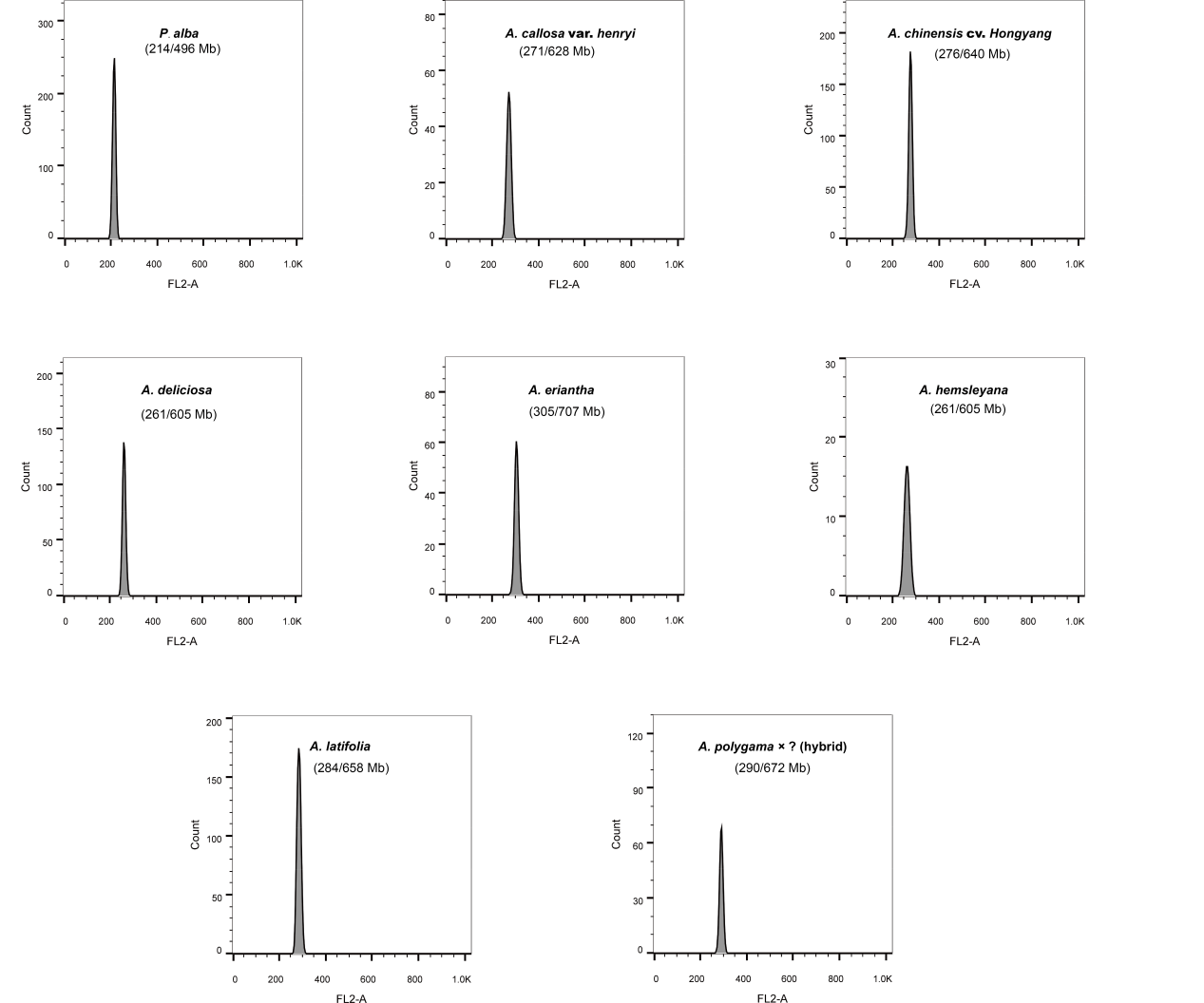


Supplementary Figure 2. Genome size of seven *Actinidia* materials by flow cytometry. Comparison of fluorescence intensity between *P. alba* (as an internal reference) and seven *Actinidia* materials and 605 - 706 Mb was estimated for seven *Actinidia* materials based on their peak values and *P. alba* genome size (496 Mb).


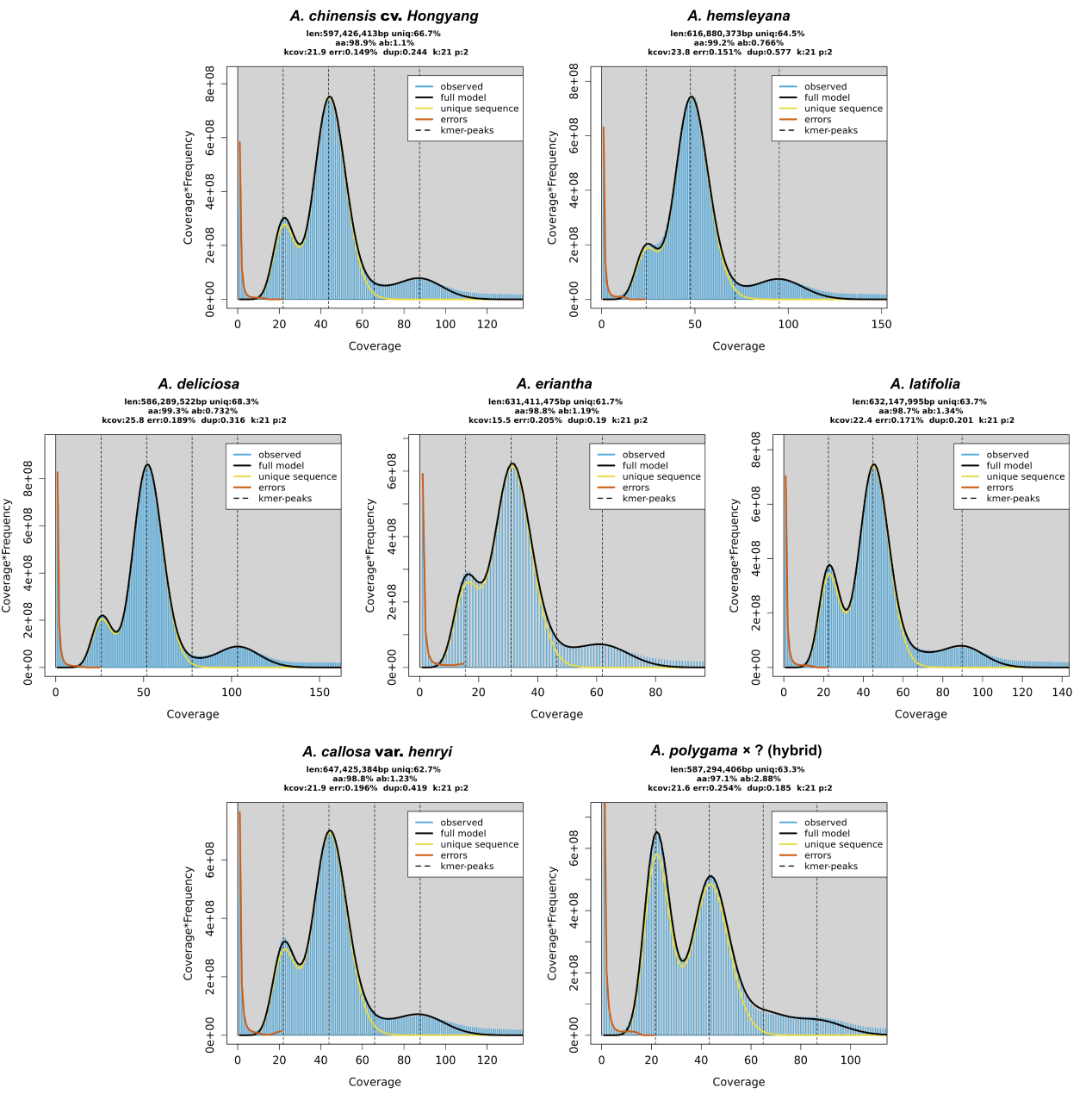


Supplementary Figure 3. Evaluation of the genome size of seven *Actinidia* materials by 21-mer analysis.


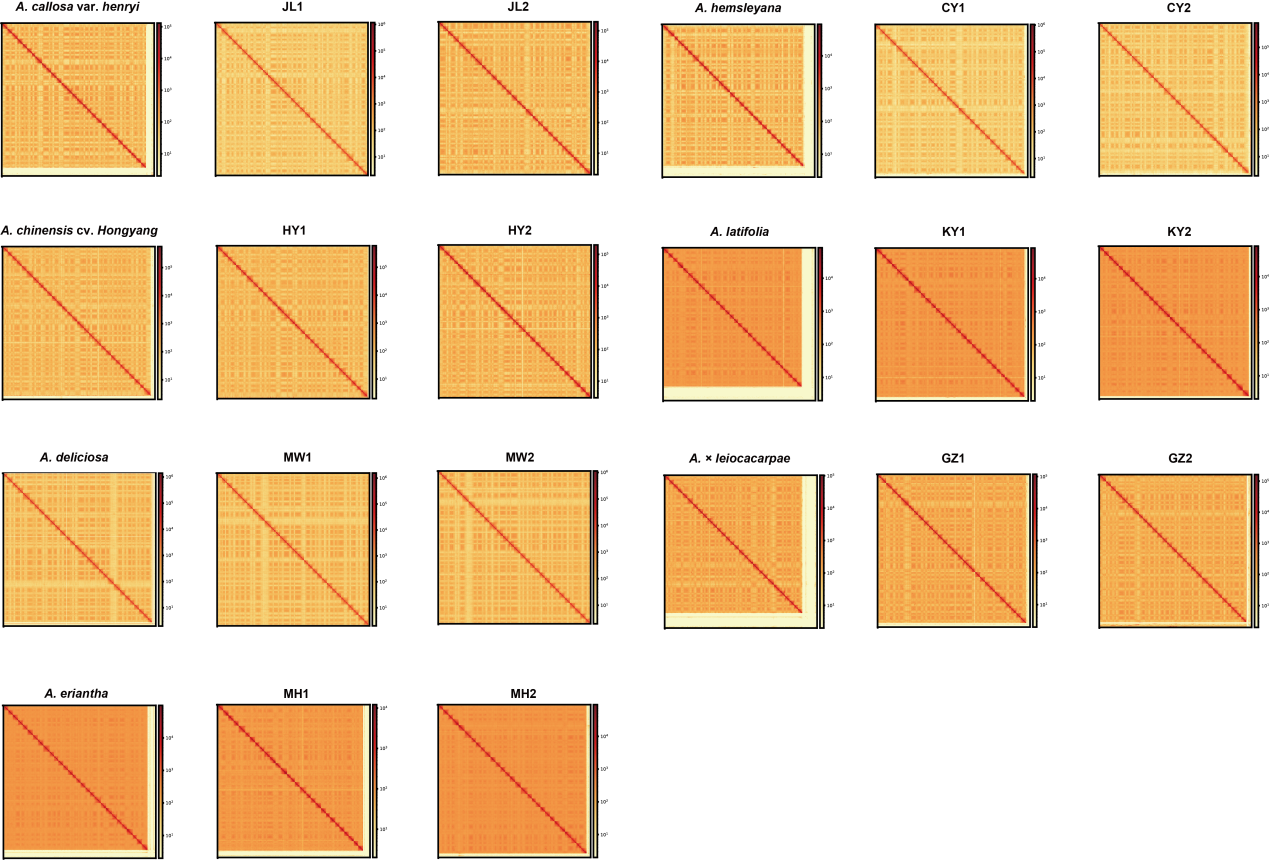


Supplementary Figure 4. Hi-C contact maps for 21 *de novo* assembled genomes. Weak interactions are indicated in yellow and strong interactions are indicated in dark red.


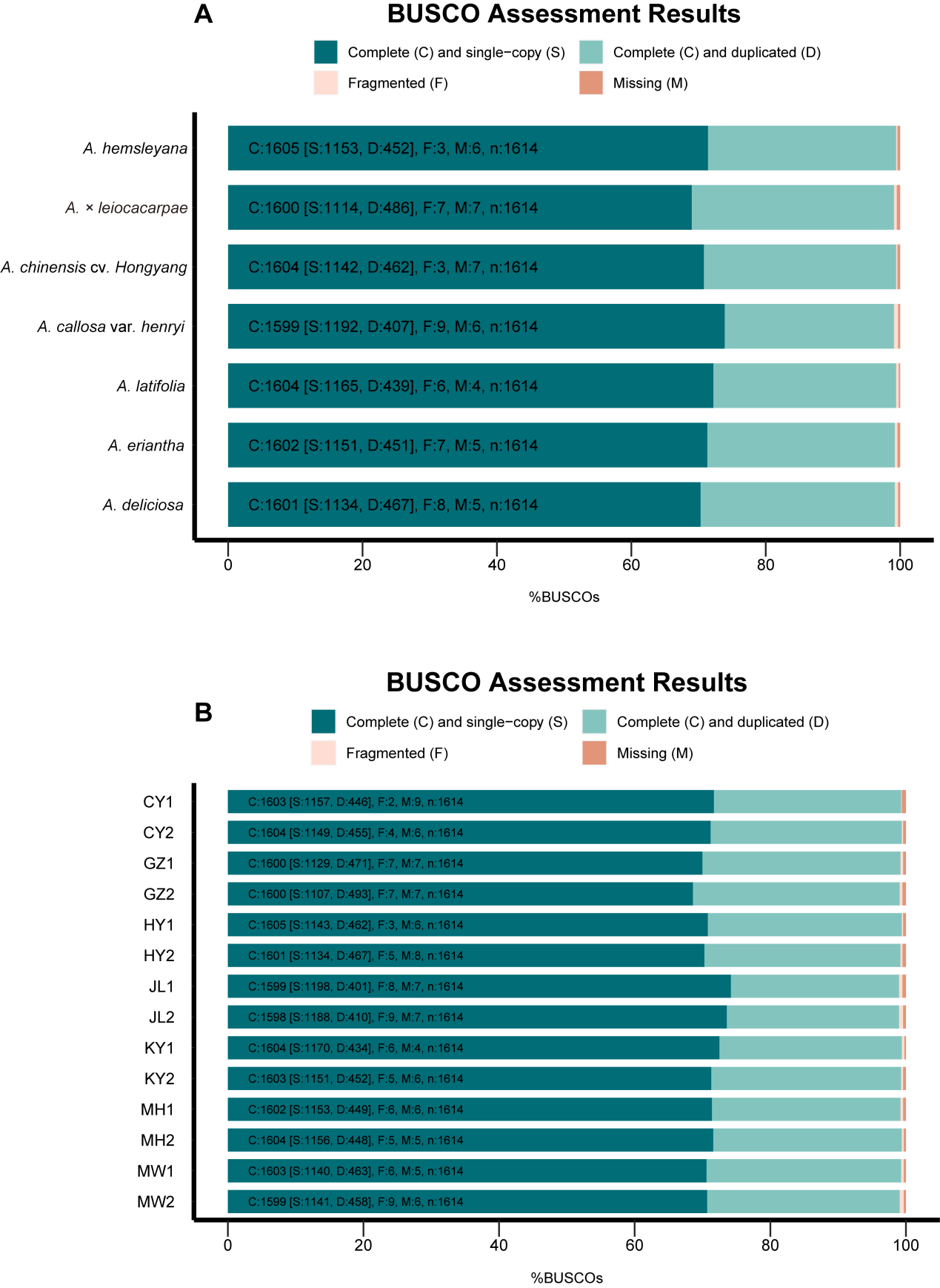


Supplementary Figure 5. Quality assessment of the A seven assembled primary genome and B 14 assembled haplotype genome using BUSCOs.


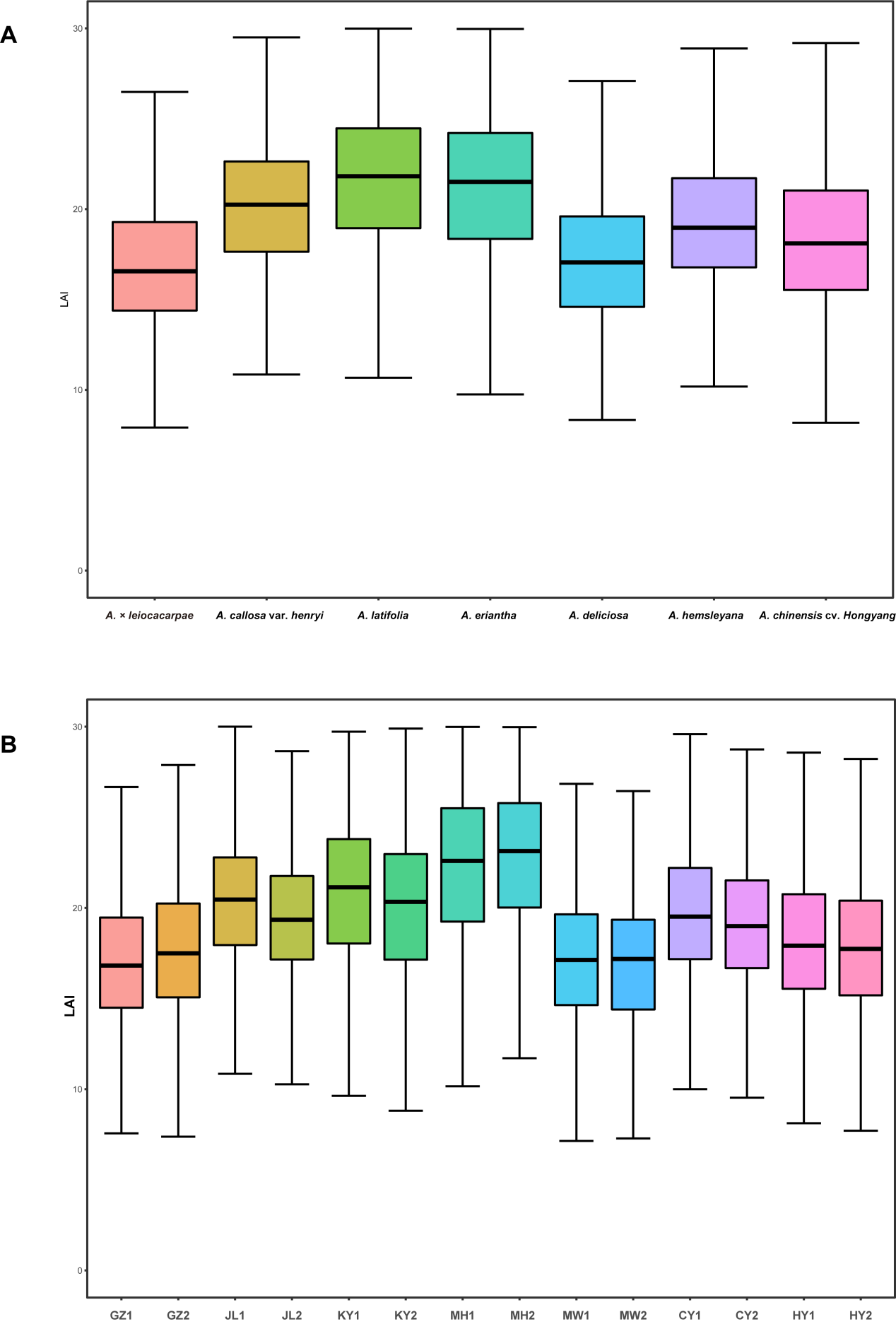


Supplementary Figure 6. Quality assessment of the A seven assembled primary genome and B 14 assembled haplotype genome using LAI.


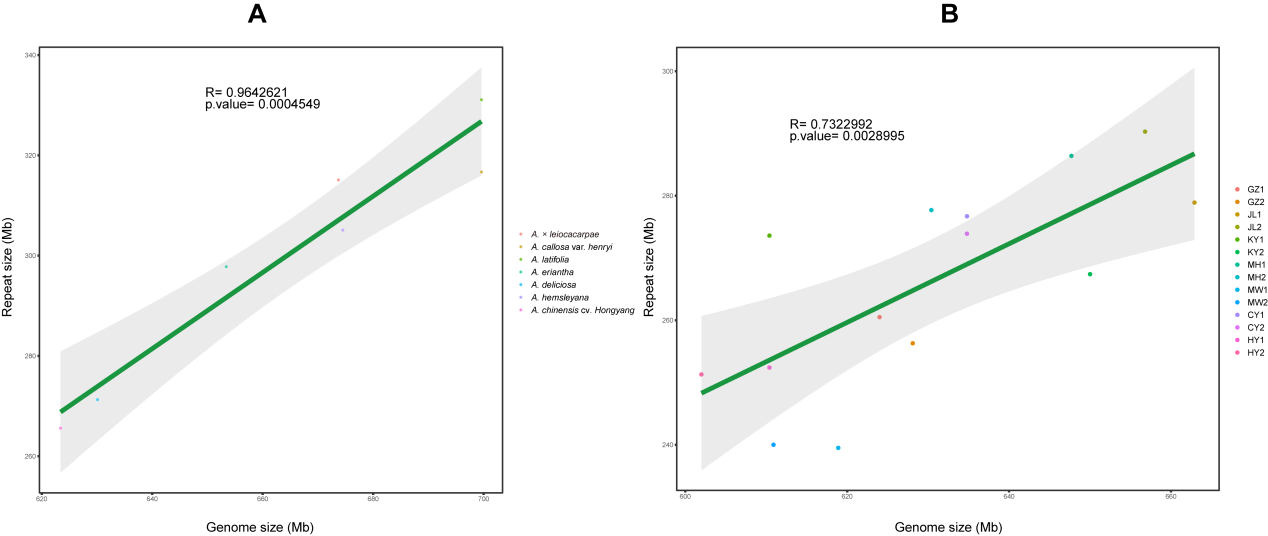


Supplementary Figure 7. Correlation analysis between repeat size and the assembly size of each A primary genome and B haplotype genome.


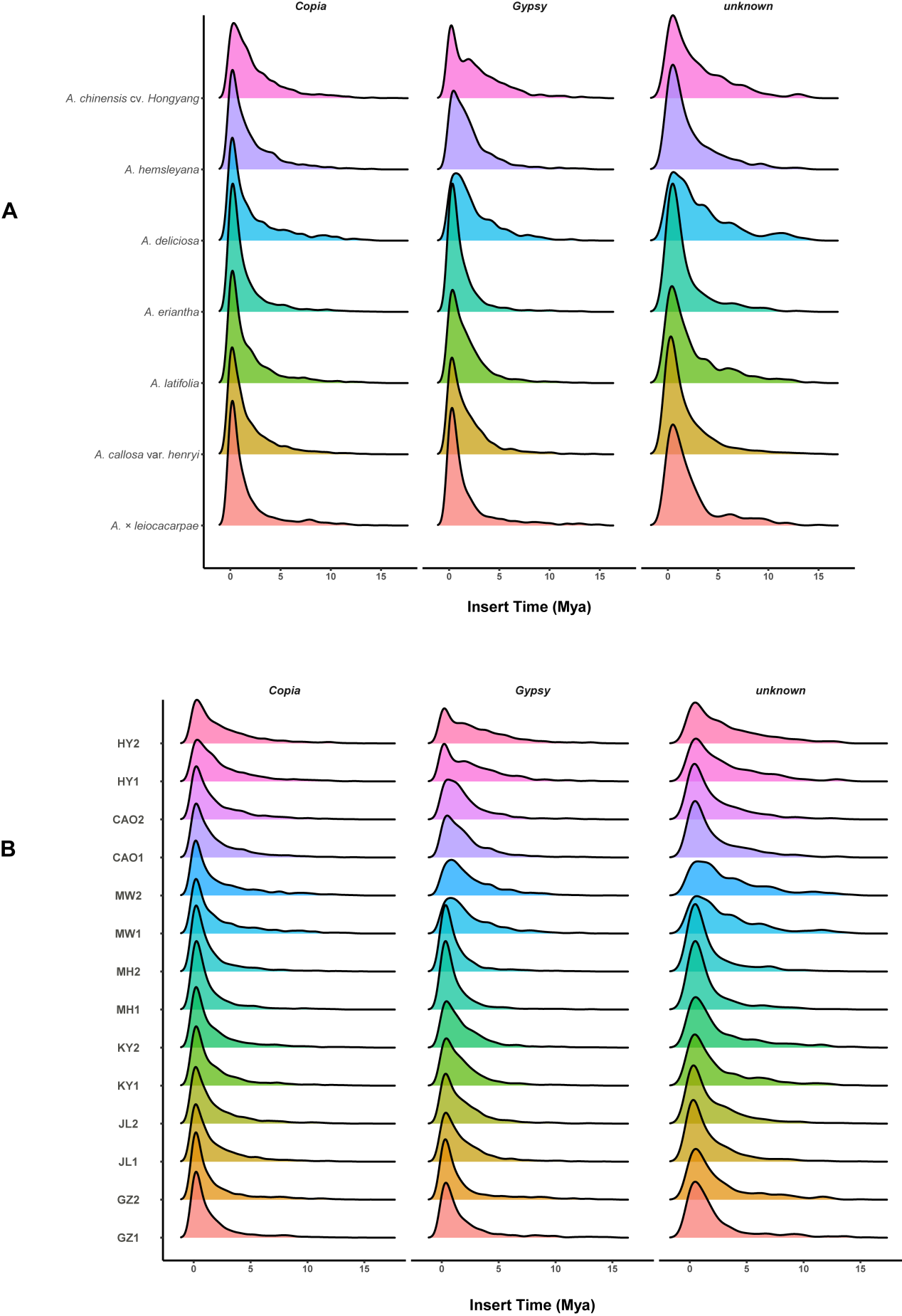


Supplementary Figure 8. The number of intact LTR-RTs birthed at different times in A 7 primary genomes and B 14 assembled haplotype genomes.


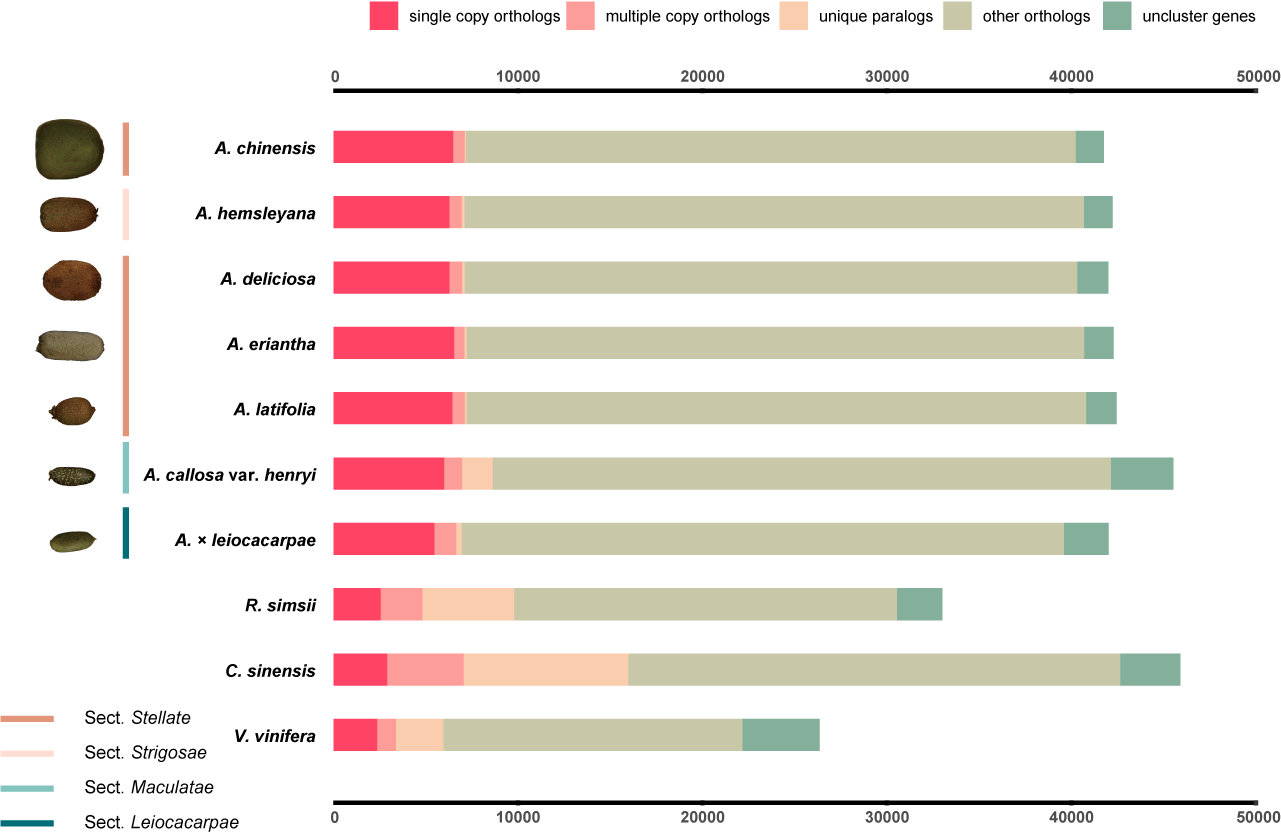


Supplementary Figure 9. Clusters of orthologous and paralogous gene families in *Actinidia* and three other plant genomes. Only the longest isoform of each gene was used. Gene families were identified using the Orthofinder package with default parameters.


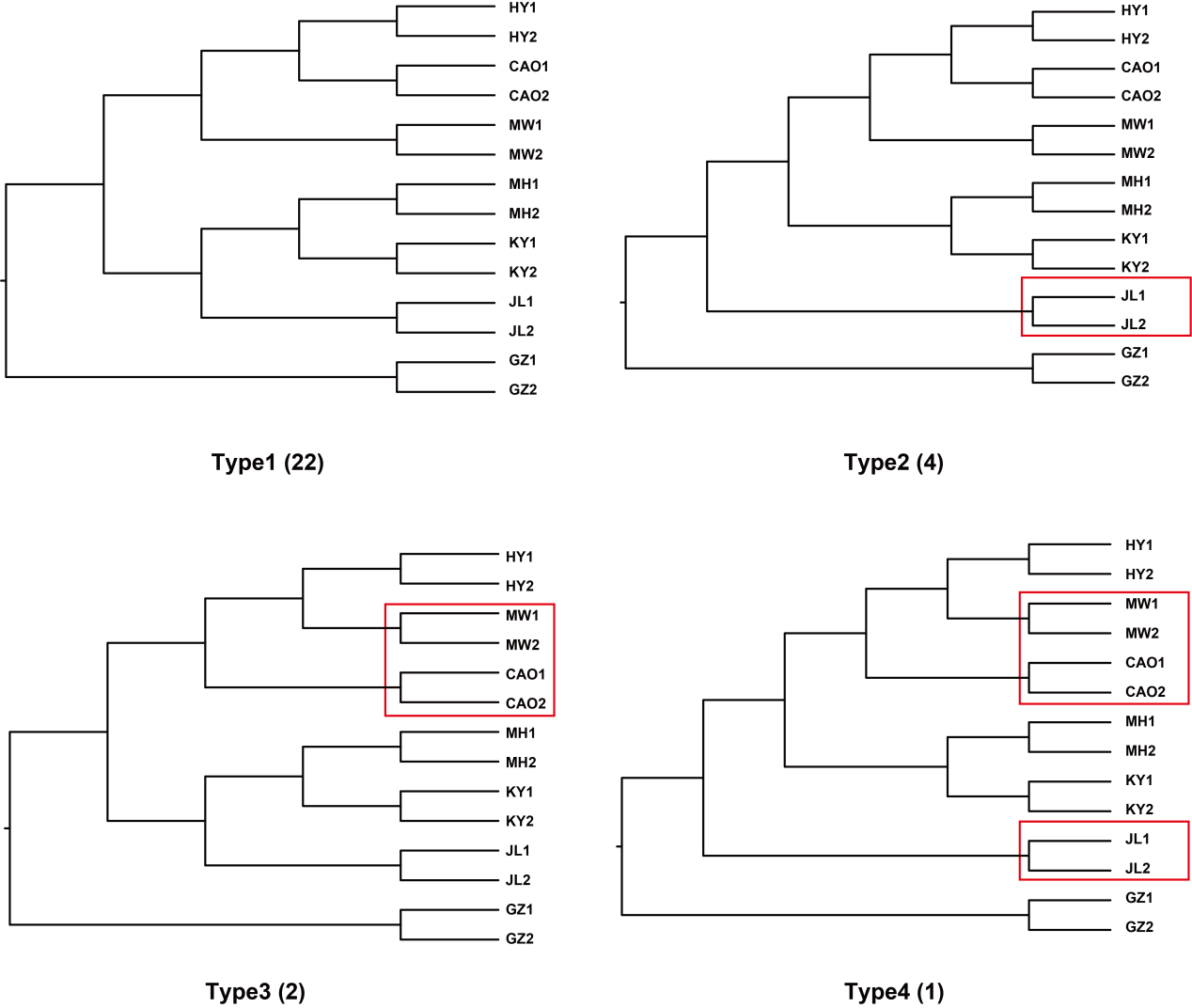


**Supplementary Figure 10.** Single-copy gene phylogenetic trees of 29 chromosomes in 14 haplotype genomes. Specific haplotype genomes are marked with red boxes to show inconsistency with the all single-copy gene phylogenetic trees.


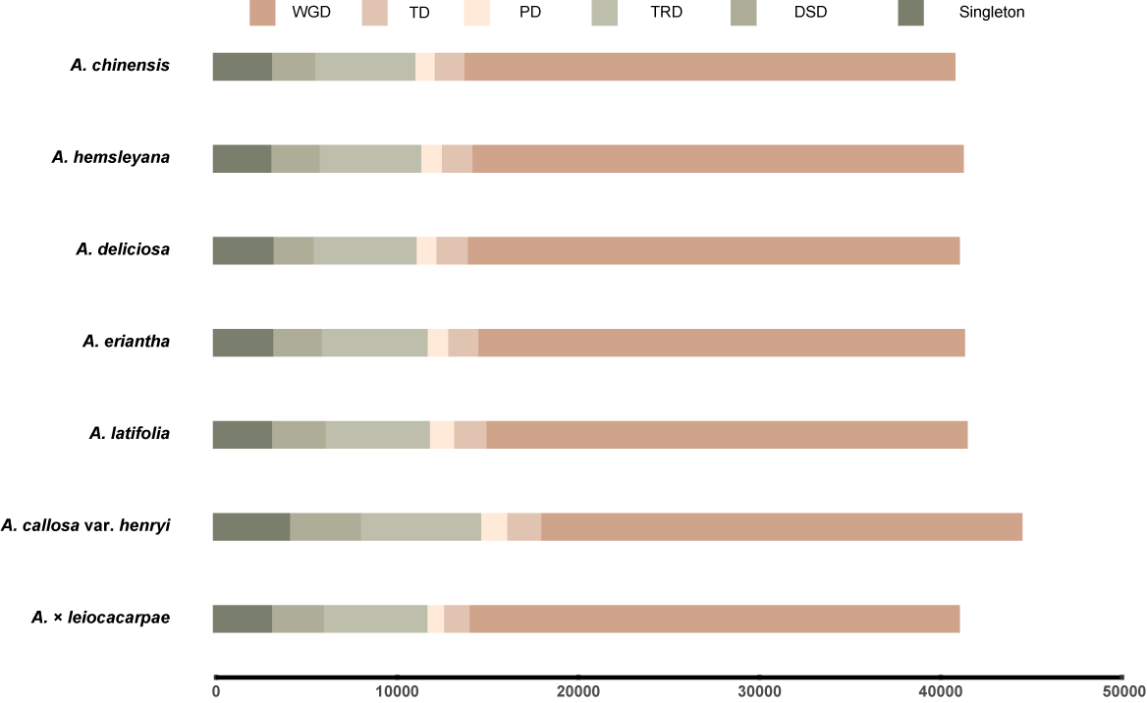


**Supplementary Figure 11.** Different gene duplication modes in seven *Actinidia* primary genomes.


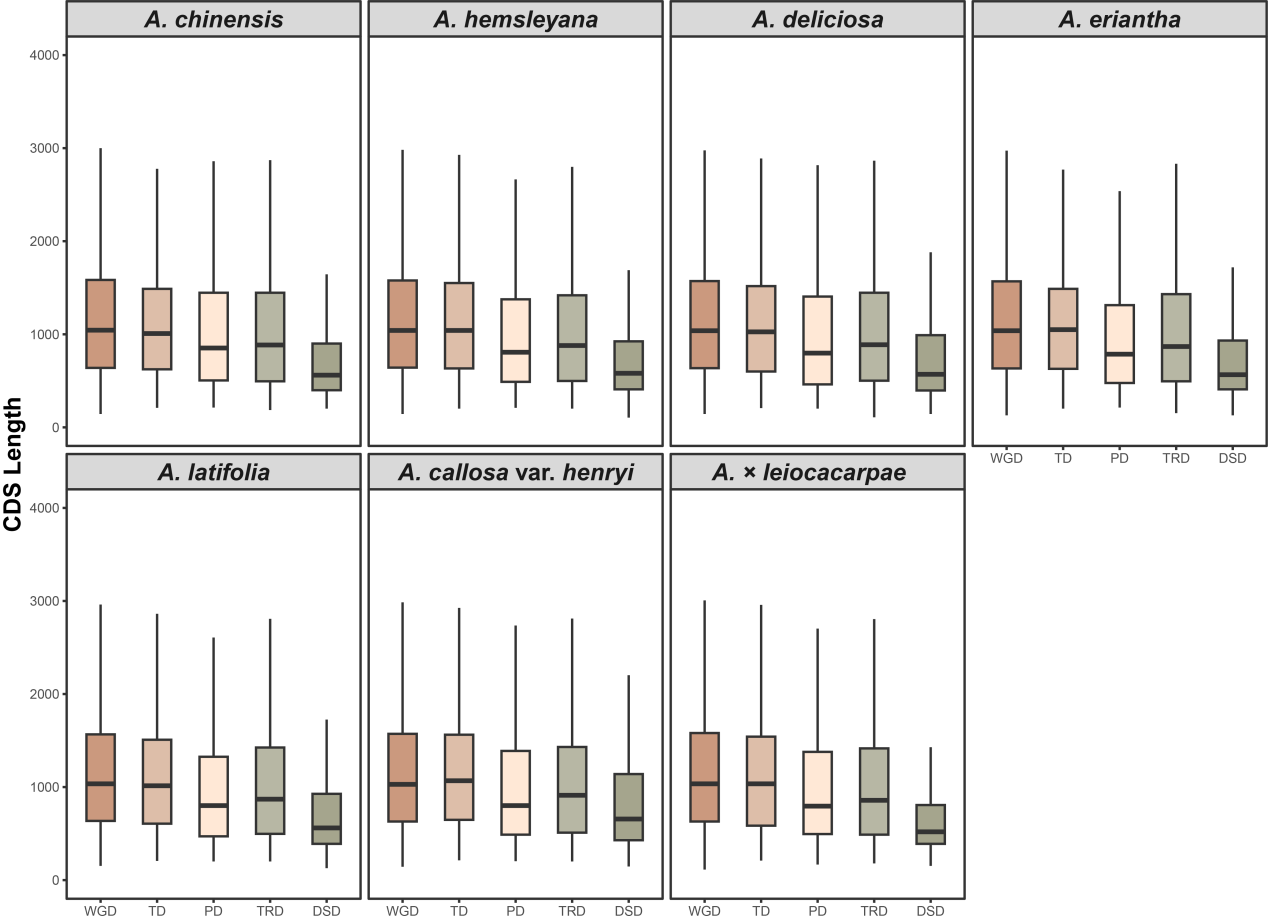


**Supplementary Figure 12.** CDS length distributions of gene pairs from duplications of seven primary genomes.


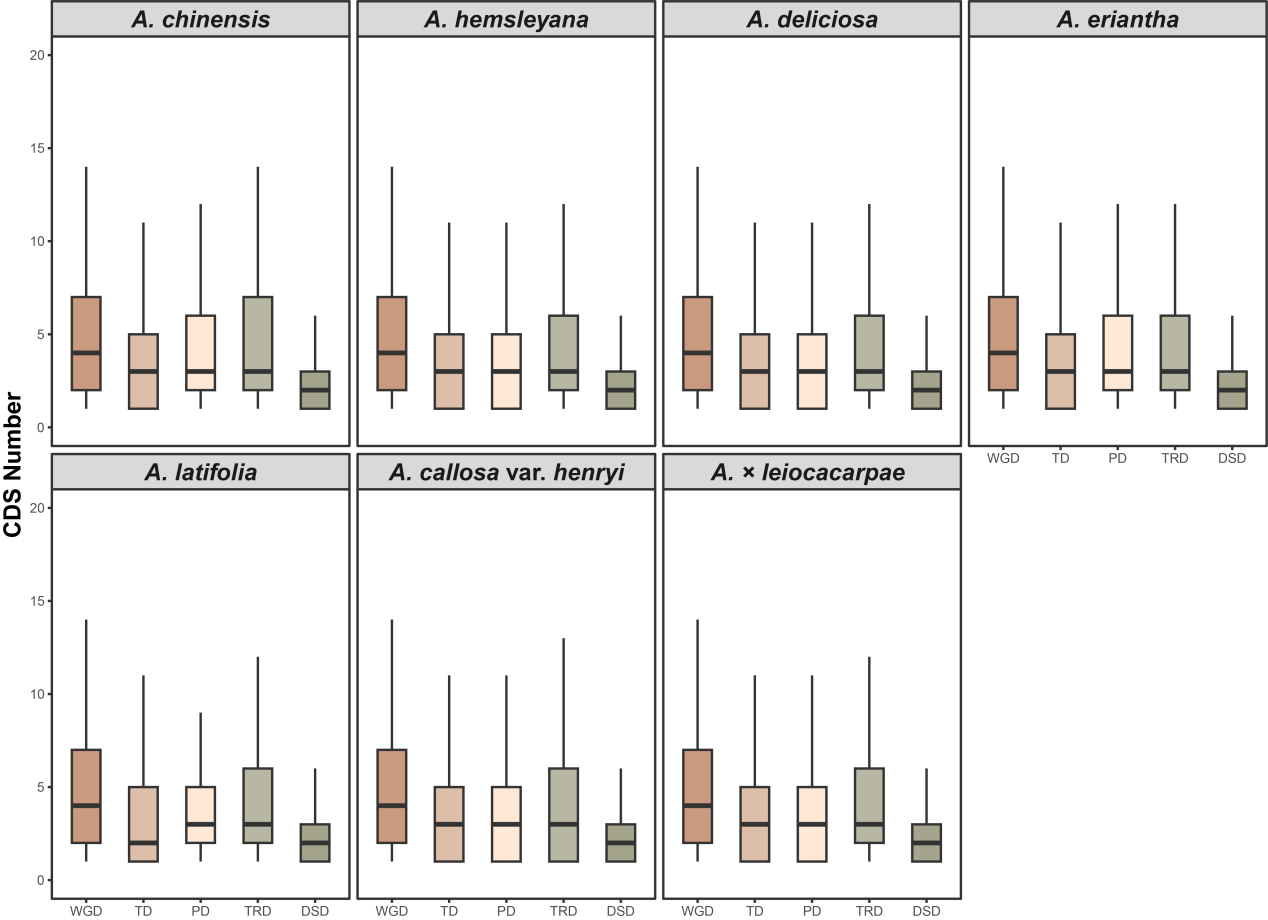


**Supplementary Figure 13.** CDS numbers of gene pairs from different duplications in seven primary genomes.


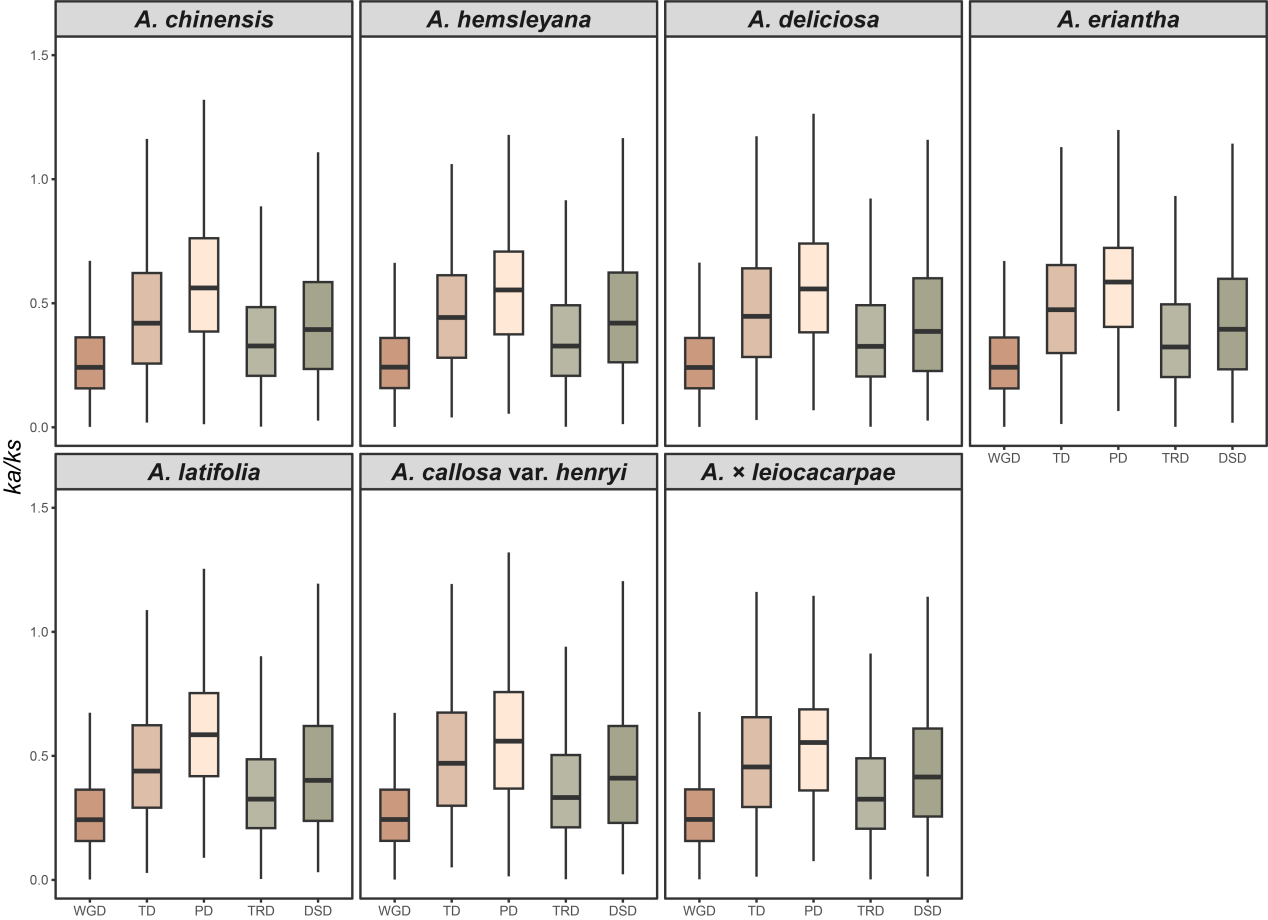


**Supplementary Figure 14.** *Ka/Ks* distributions of gene pairs from different duplication modes in seven primary genomes.


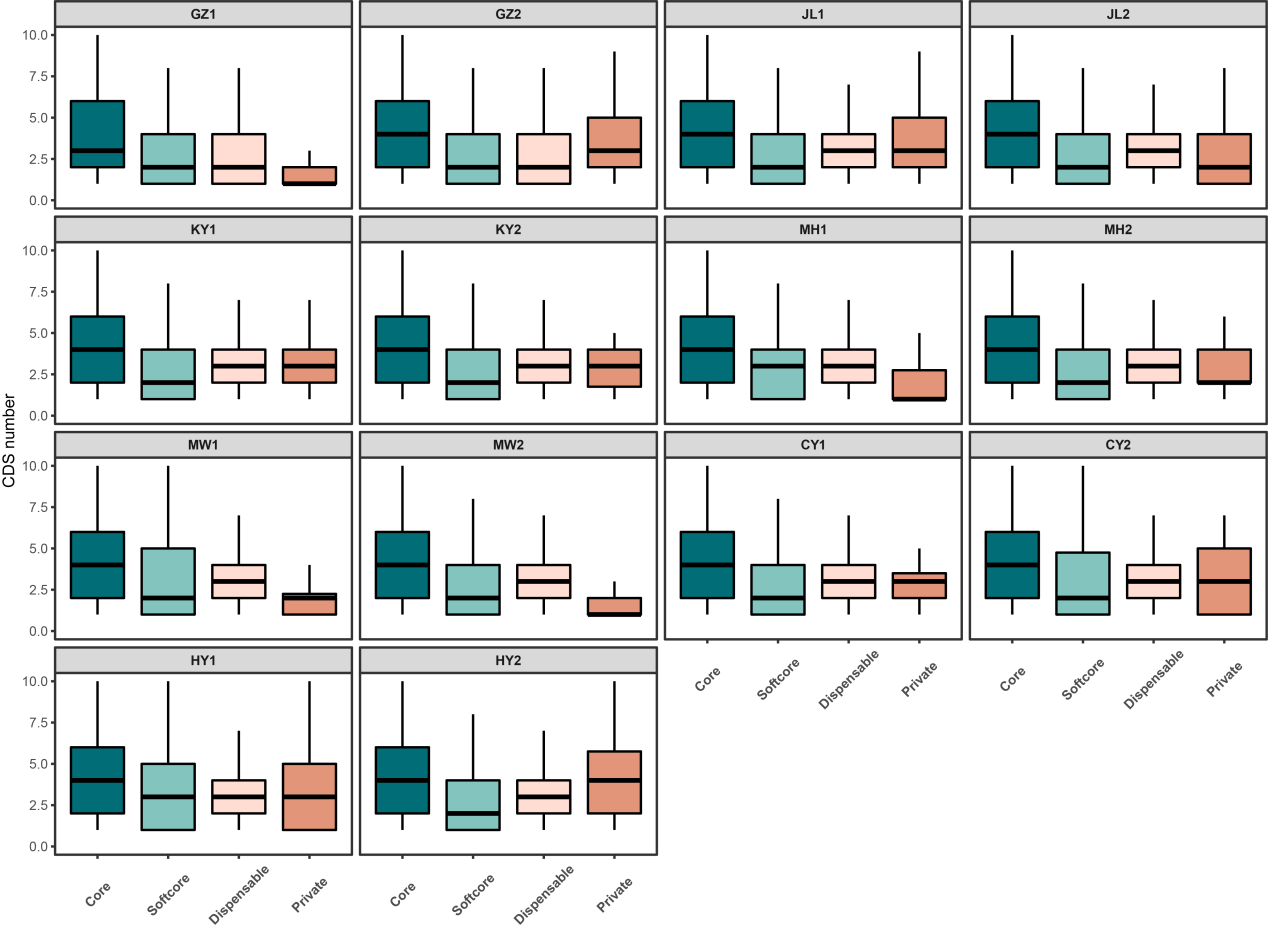


**Supplementary Figure 15.** CDS numbers of core, softcore, dispensable and private genes in 14 haplotype genomes.


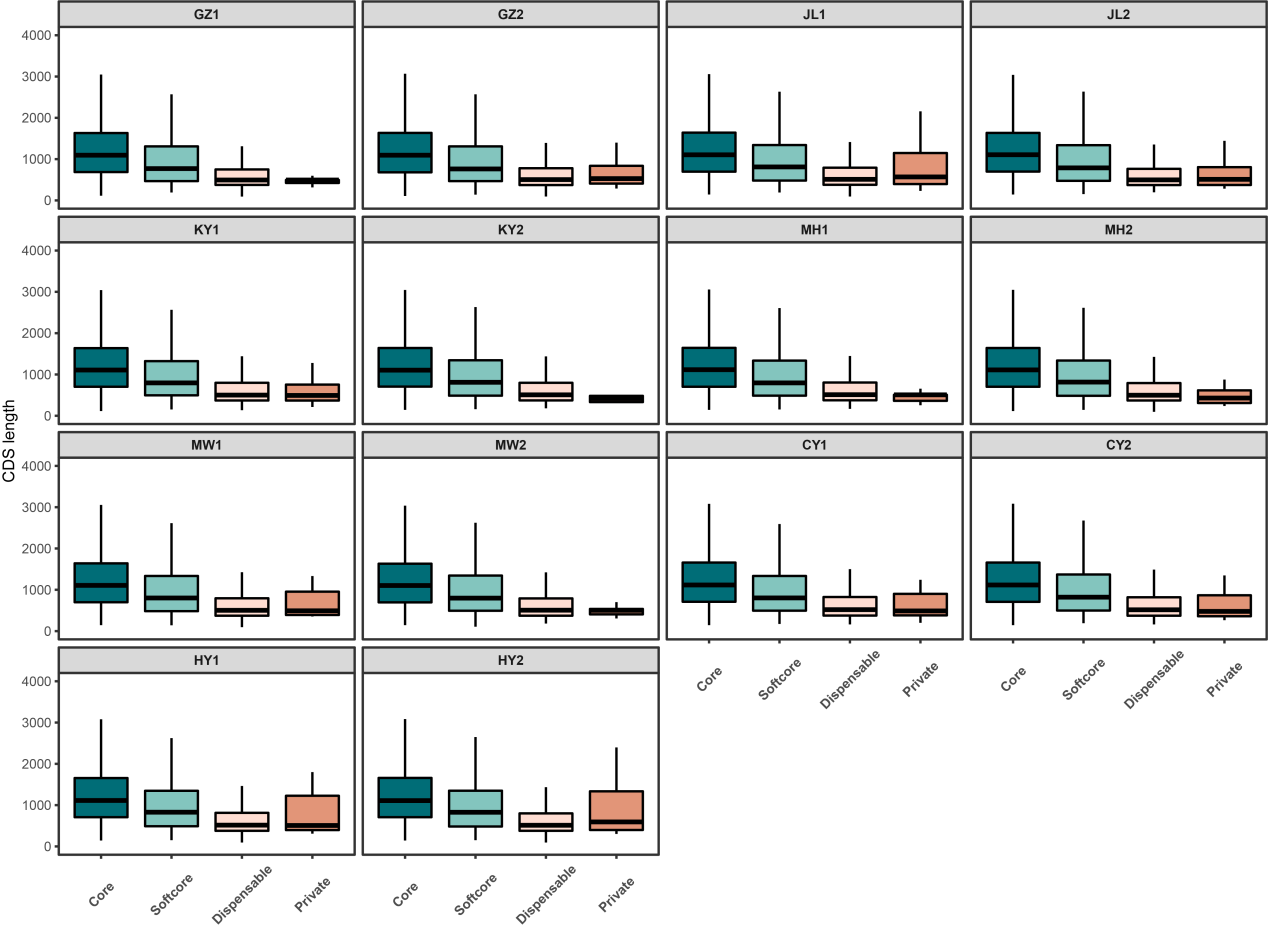


**Supplementary Figure 16.** CDS lengths of core, softcore, dispensable and private genes in 14 haplotype genomes.


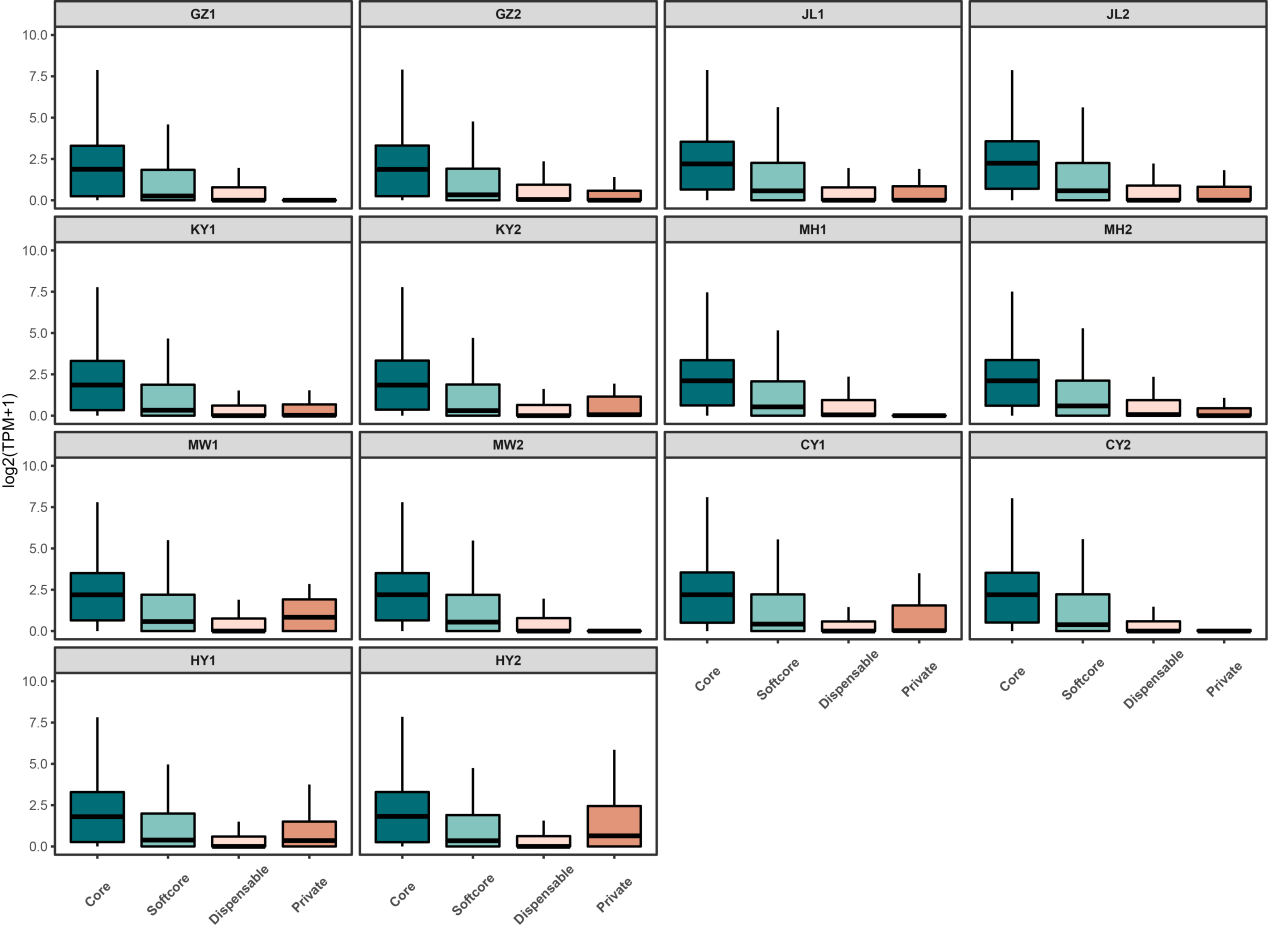


**Supplementary Figure 17.** Leaf expressions of core, softcore, dispensable and private genes in 14 haplotype genomes.


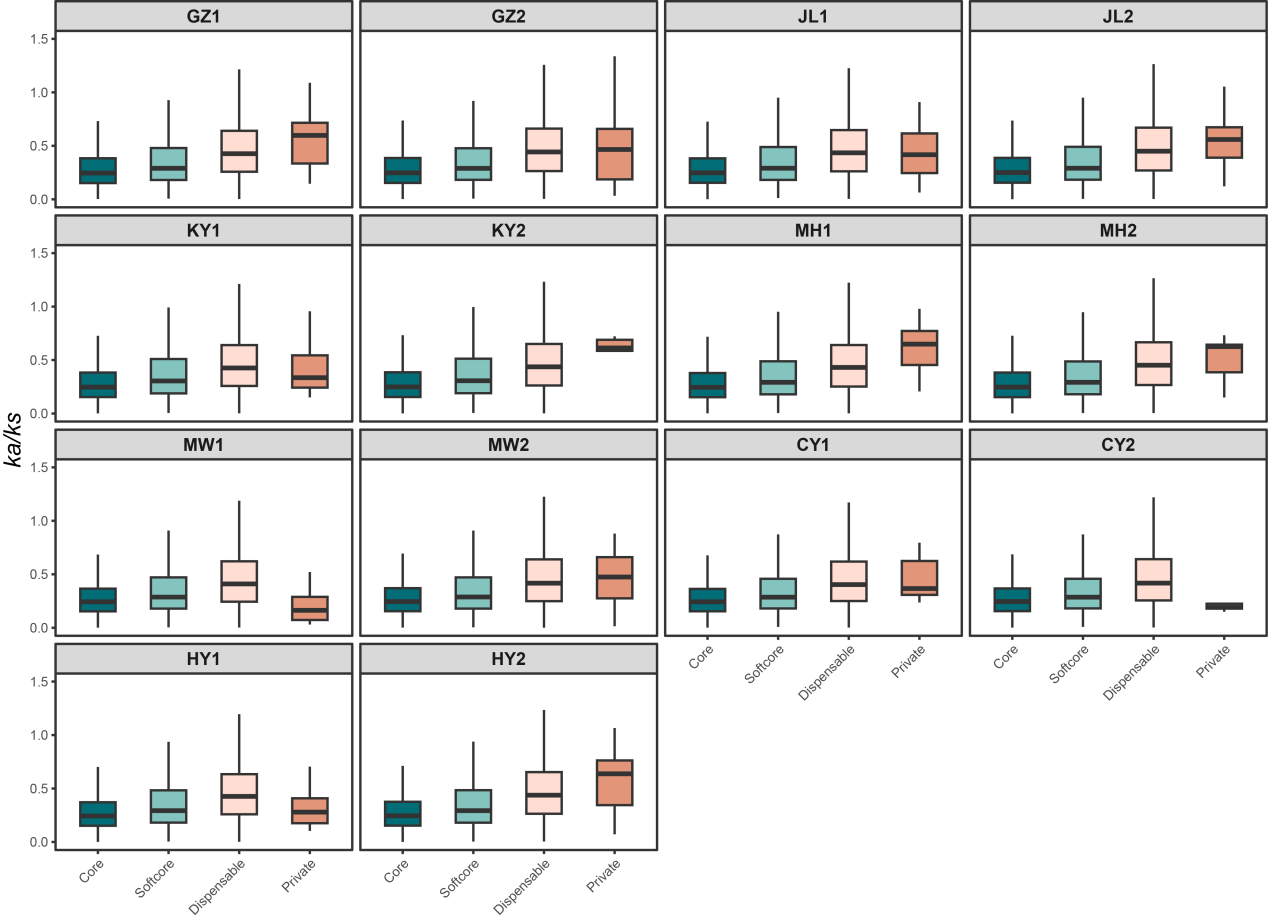


**Supplementary Figure 18.** Selective constraints of core, softcore, dispensable and private genes in 14 haplotype genomes.


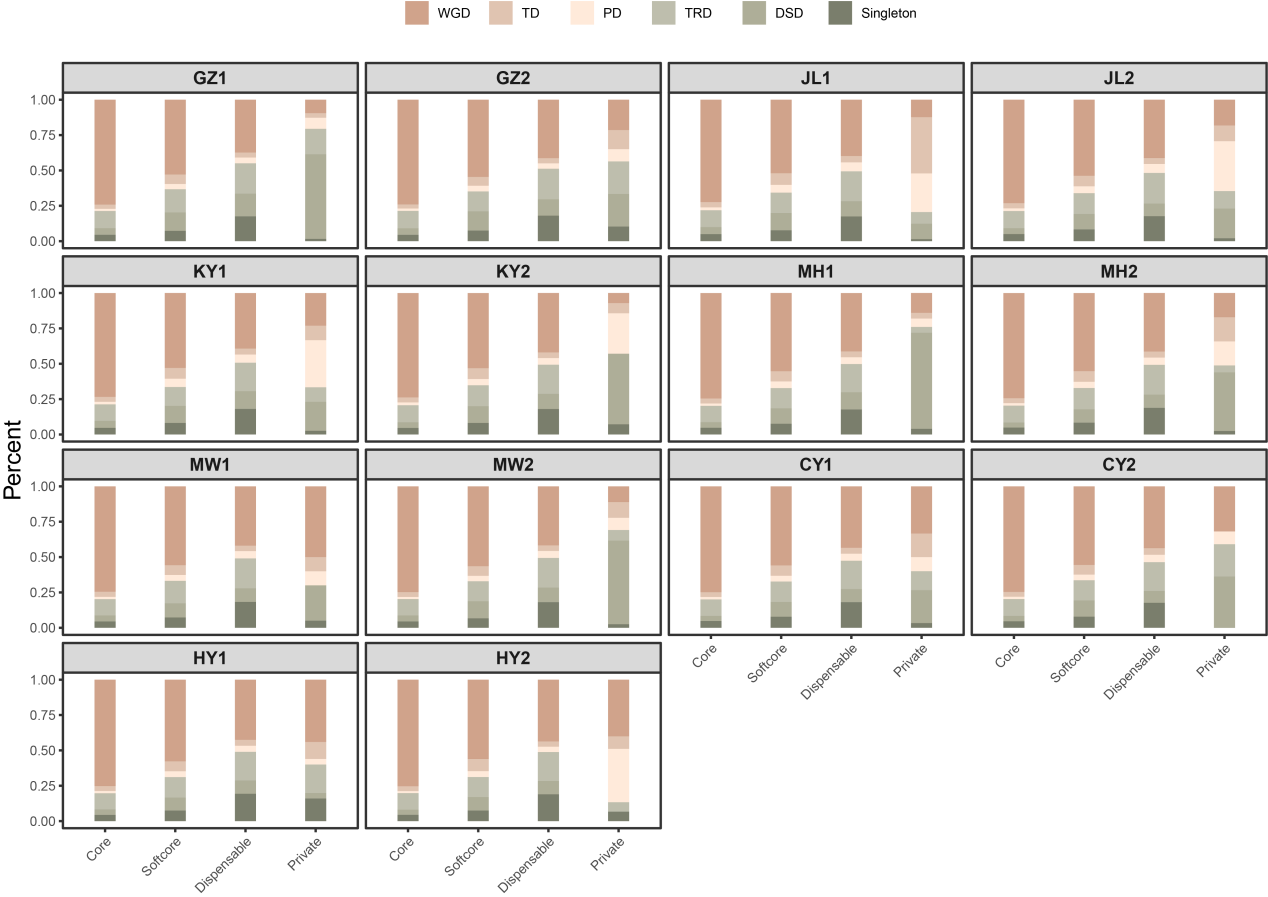


**Supplementary Figure 19.** Overall proportions of singleton, DSD, TRD, PD, TD and WGD genes in 14 haplotype genomes based on their pan-genome classification.


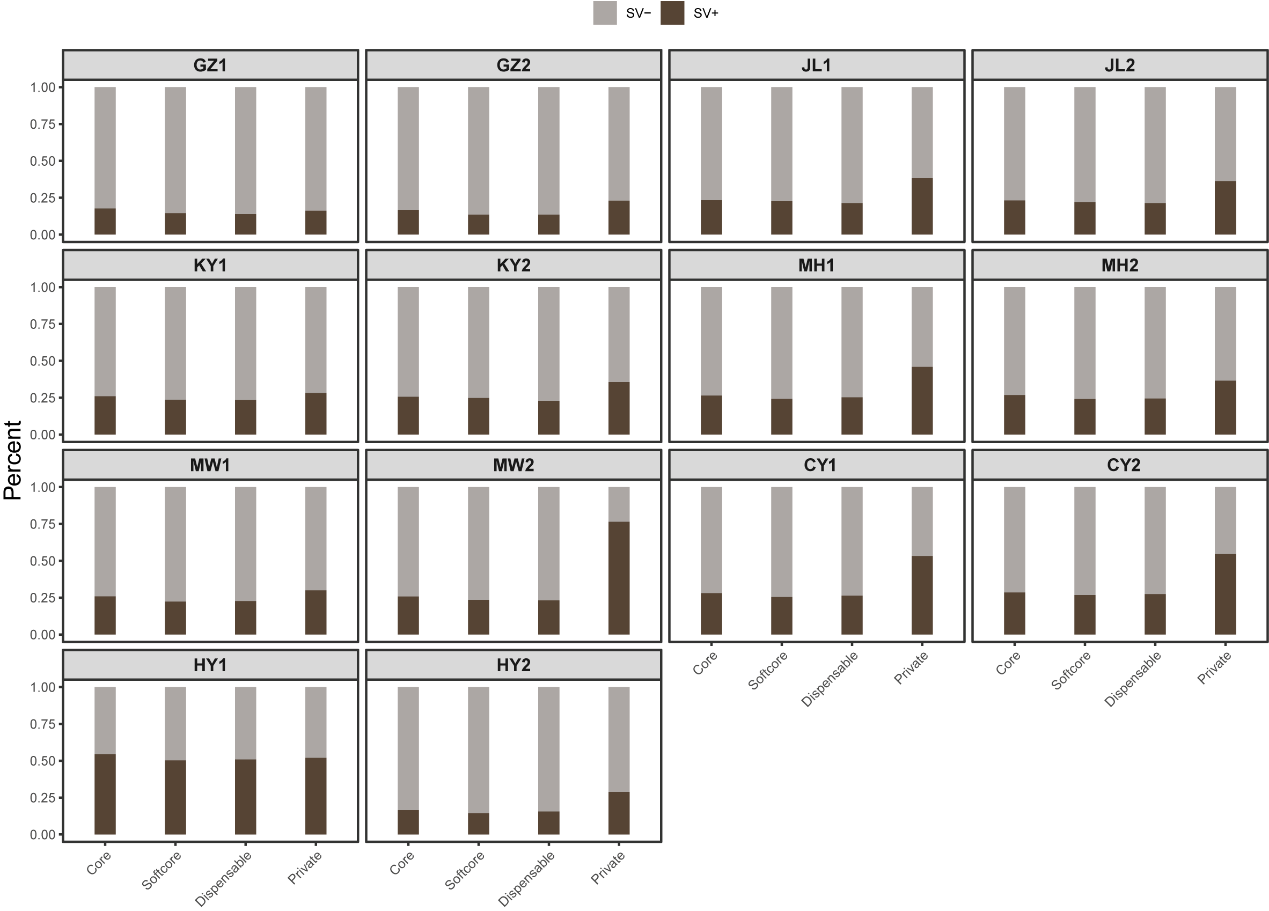


**Supplementary Figure 20.** Overall proportions of genes with SVs and without SVs in 14 haplotype genomes based on their pan-genome classification.


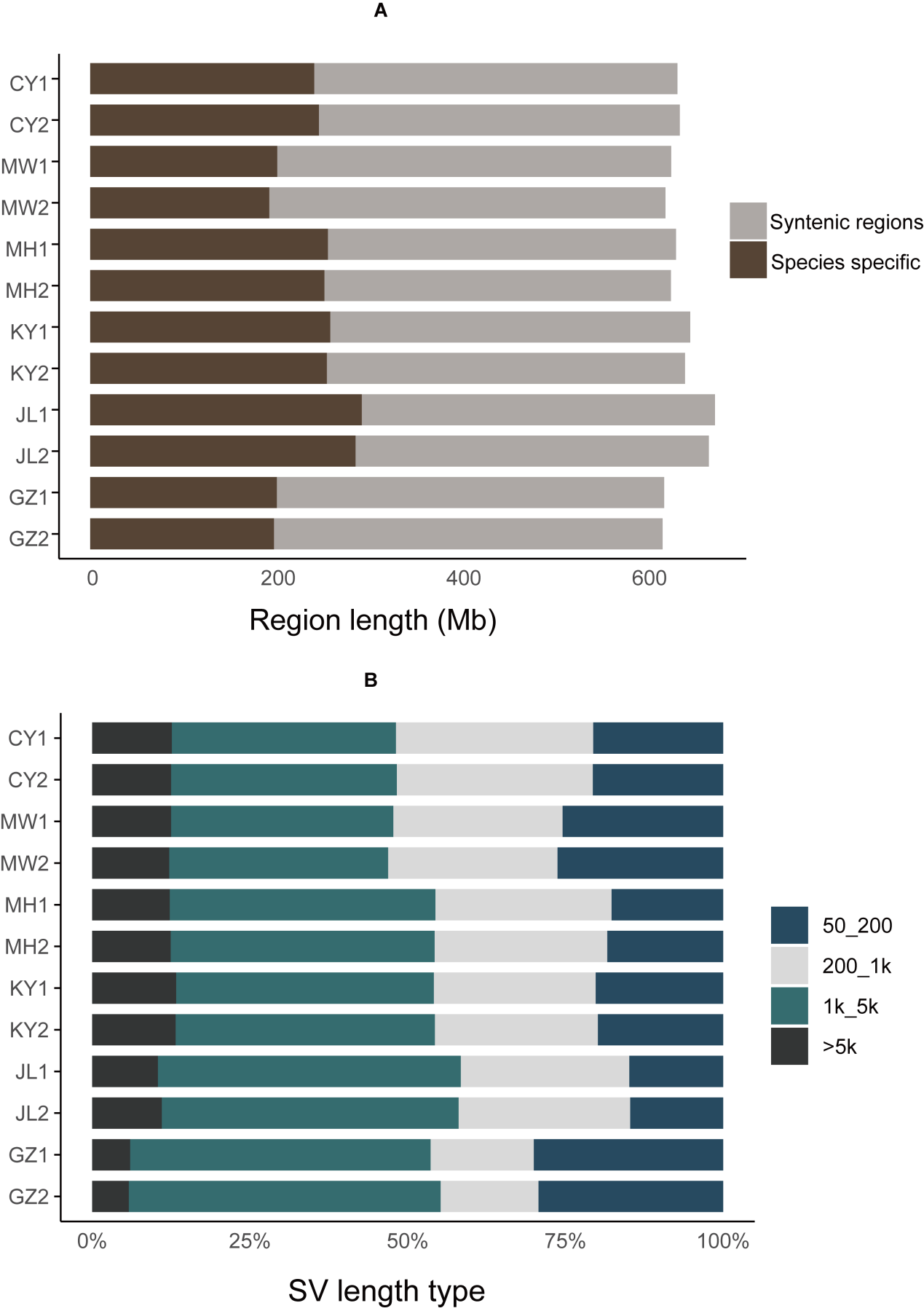


**Supplementary Figure 21.** SVs detected in 12 assembled haplotype genomes. **A** species specific region; **B** SV length distribution.


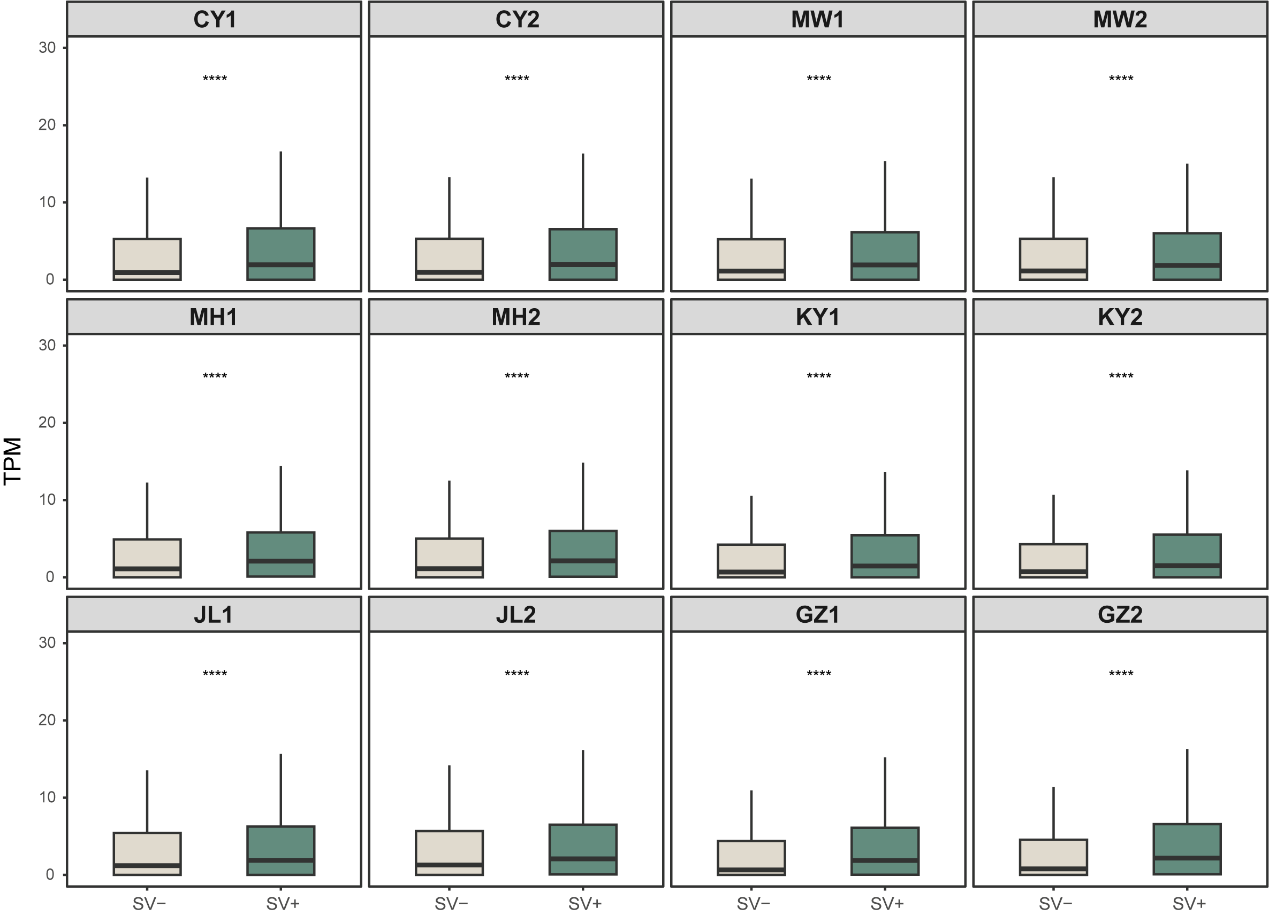


**Supplementary Figure 22.** The expression levels of leaf genes with or without SVs in 12 haplotype genomes.


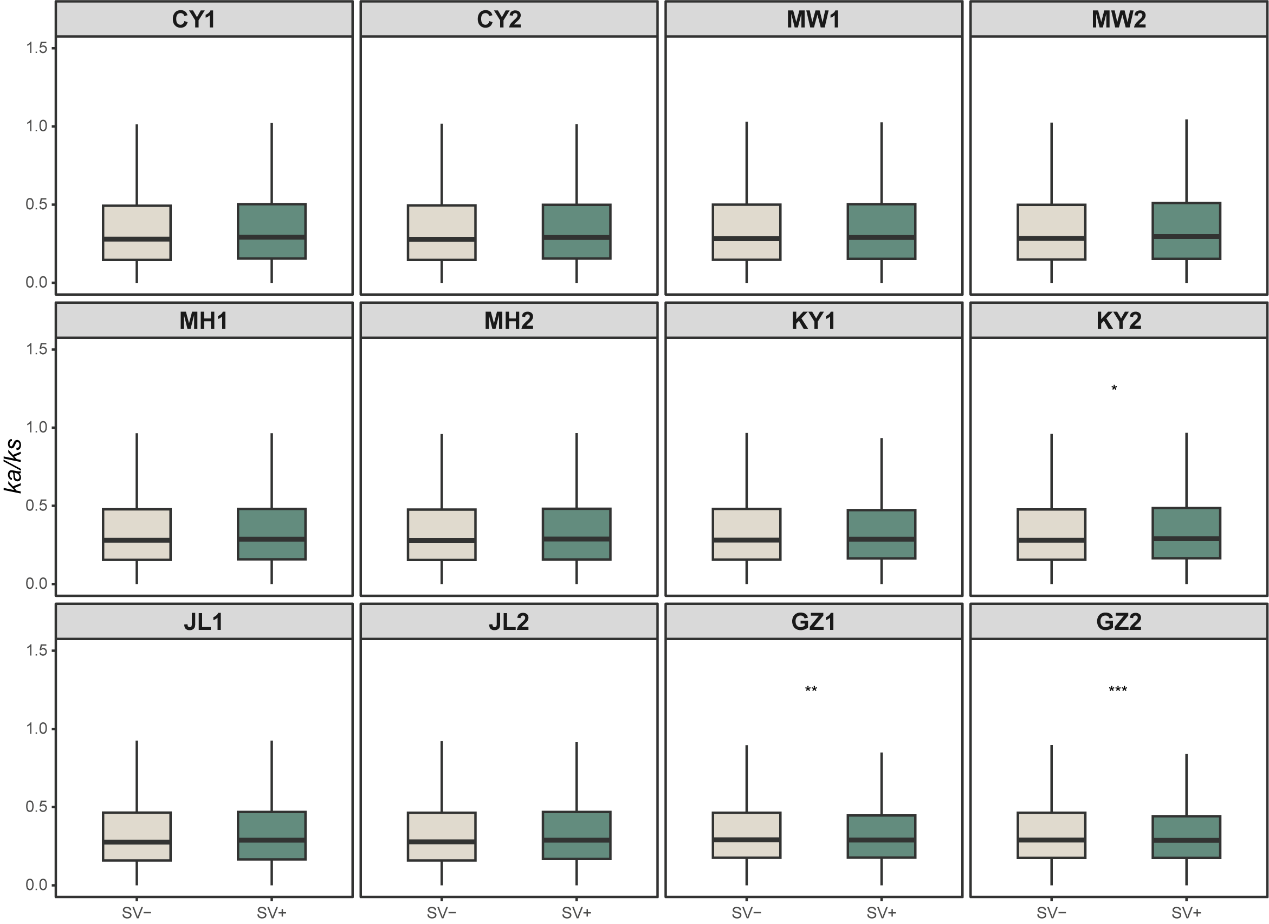


**Supplementary Figure 23.** The selective constraint of genes with or without SVs in 12 haplotype genomes.


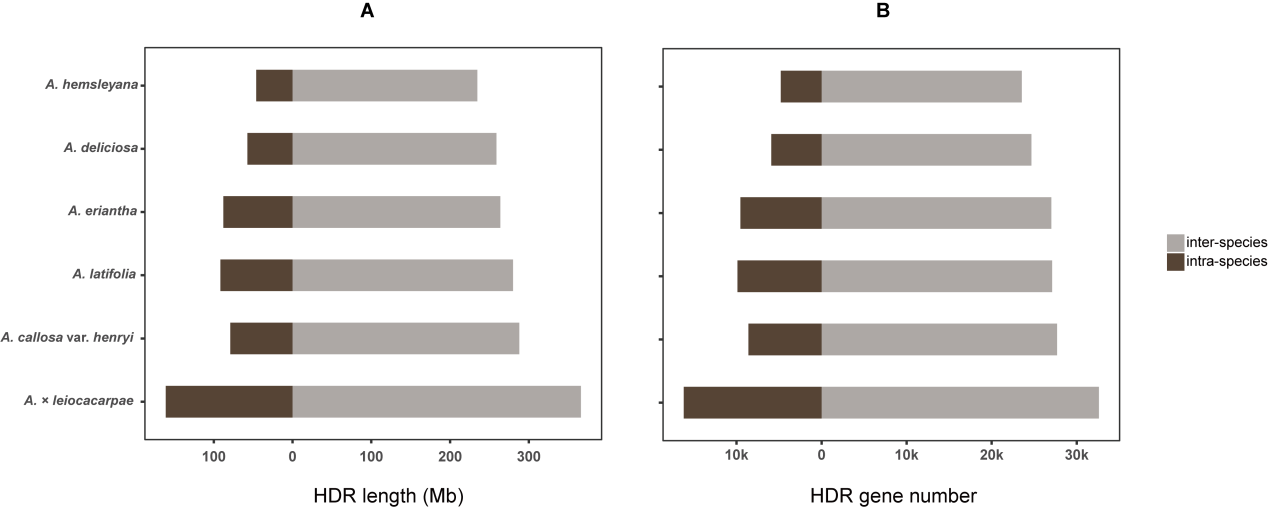


**Supplementary Figure 24.** Comparison of HDR between and within materials for six haplotype genomes. **A** HDR length, **B** number of genes located in HDR.


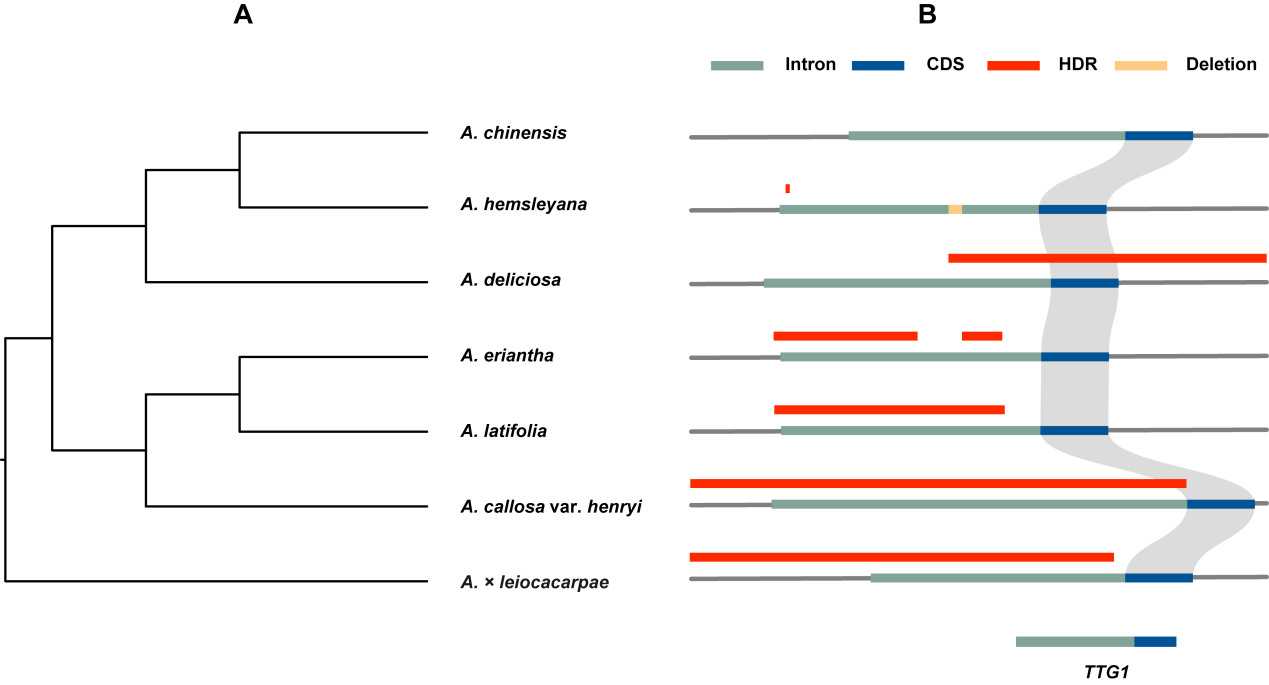


**Supplementary Figure 25. A** The phylogenetic tree of seven primary genomes. **B** The landscape of variations of *TTG1* gene in seven genomes. Syntenic regions, CDS regions, intron regions, HDR and SVs (Deletion) are indicated in different colors: Syntenic regions (grey), CDS regions (blue), intron regions (green), HDR (red) and Deletion (yellow).


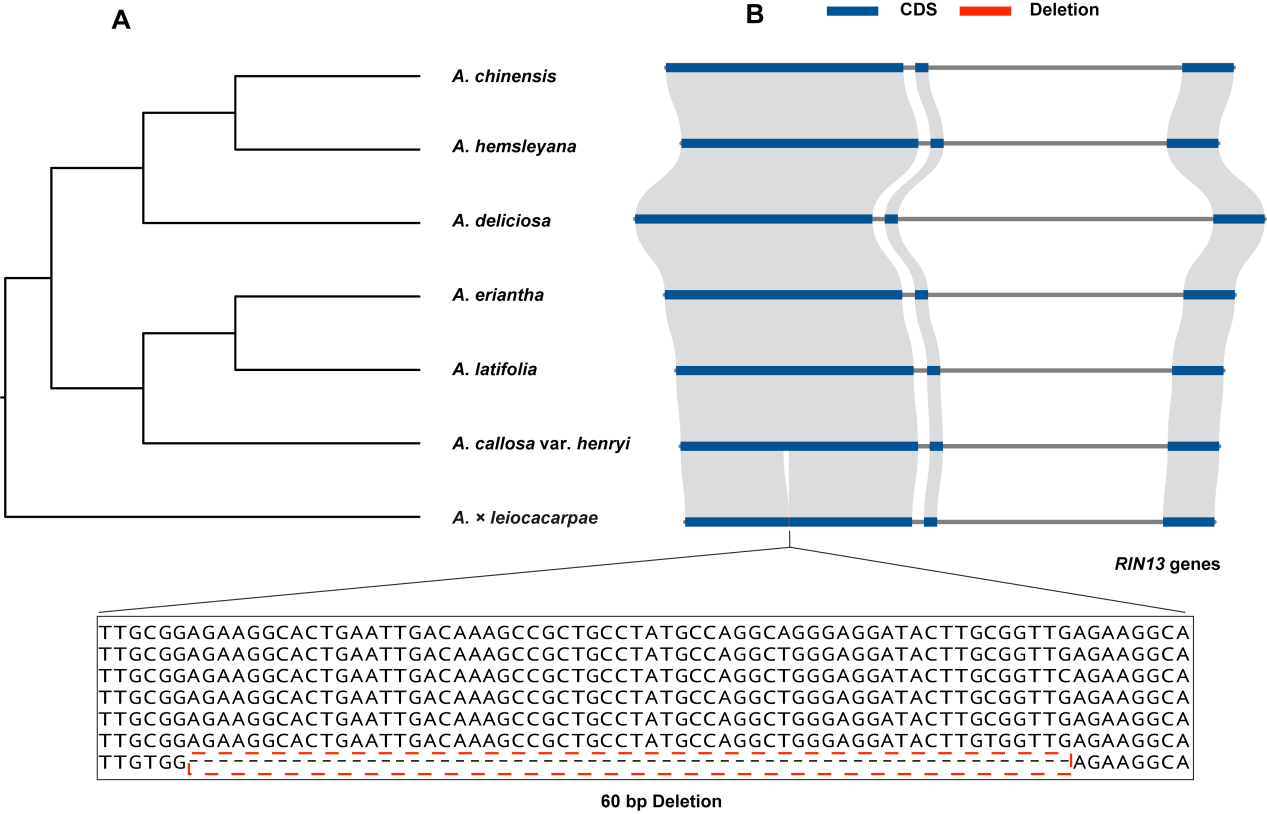


**Supplementary Figure 26. A** The phylogenetic tree of seven primary genomes of *Actinidia*. **B** The landscape of variations of *RIN13* gene in seven genomes. Syntenic regions, CDS regions and SVs (Deletion) are indicated in different colors: Syntenic regions (grey), Deletion (red) and CDS regions (blue).


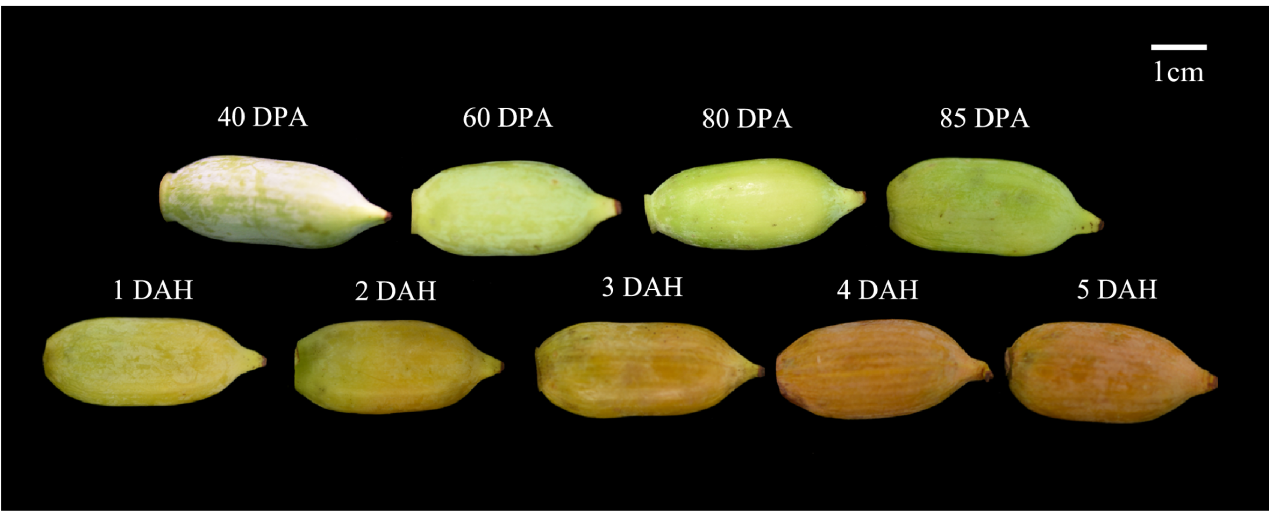


**Supplementary Figure 27.** Different staging of *A. × leiocacarpae* fruit development. DPA: days post-anthesis; DAH: days after harvest.


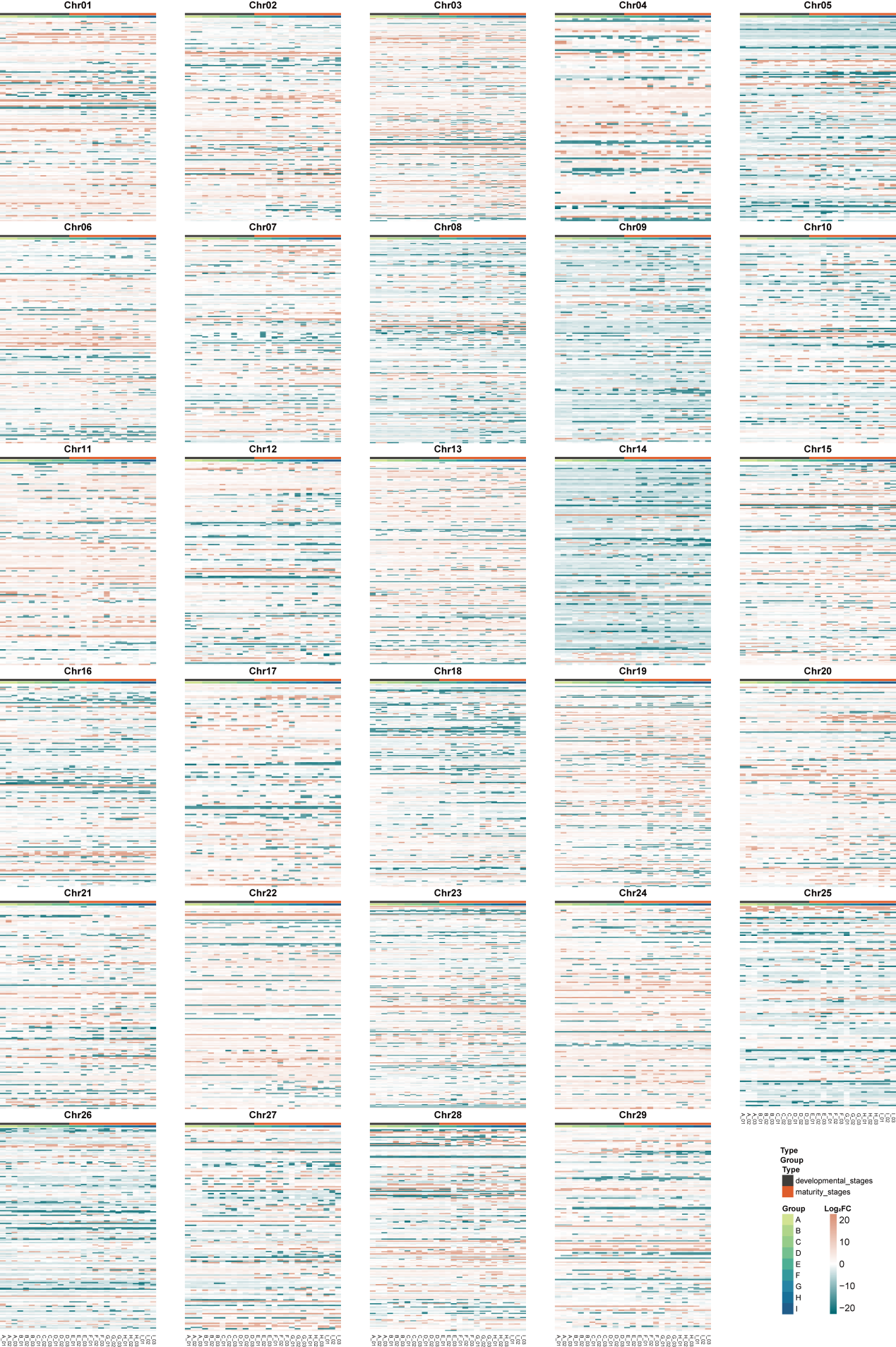


**Supplementary Figure 28.** Expression levels (log2FC) of ASE genes across the 9 stages of fruit development in *A. × leiocacarpae*. Dominance expression pattern of ASEs in GZ1 and GZ2 are colored in agate green and orange, respectively. Four developmental stages (A-D) and five maturity stages (E-I) are colored in black and red, respectively. A - 40 DPA, B - 60 DPA, C - 80 DPA, D - 85 DPA, E - 1 DAH, F - 2 DAH, G - 3 DAH, H - 4 DAH, I - 5 DAH. “_1”, “_2” and “_3” represent different biological duplicates.


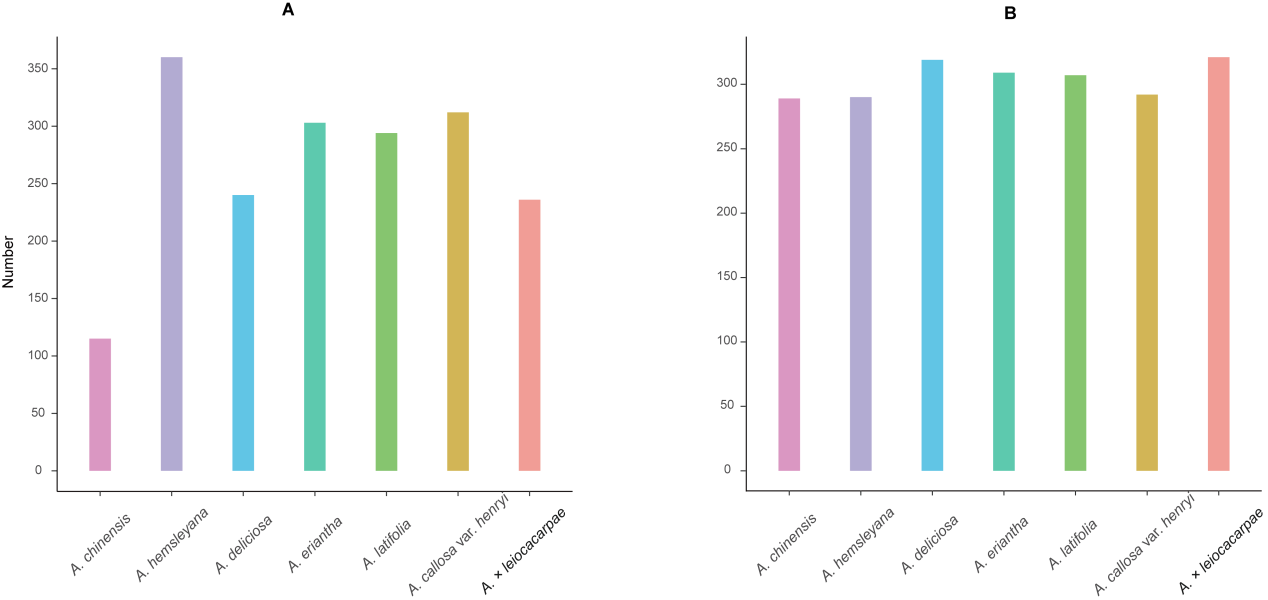


**Supplementary Figure 29.** Disease-resistance genes in 7 *Actinidia* material genomes: **A** NBS-LRR and **B** RLK-LRR.


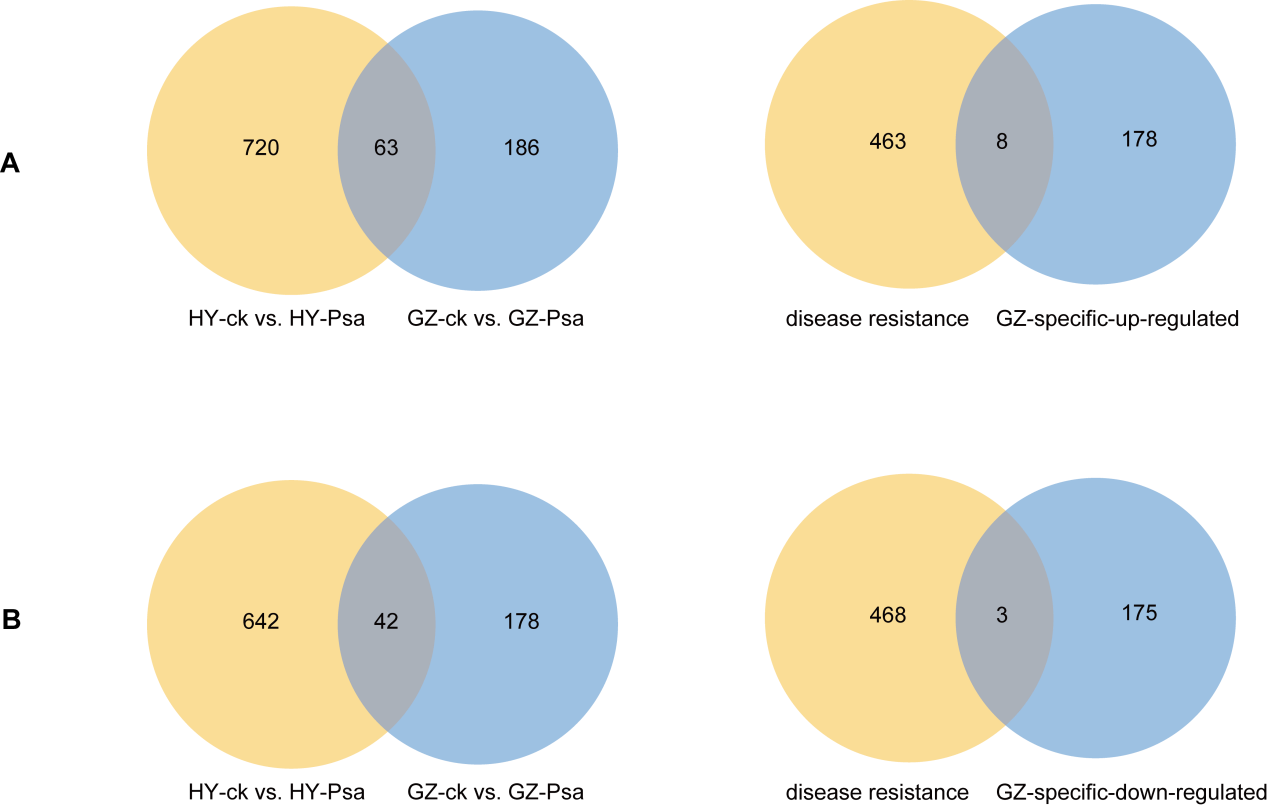


**Supplementary Figure 30.** Venn diagrams of differential expression genes and dissease resistance genes in *A. × leiocacarpae* (GZ) and *A. chinensis* cv. ‘Hongyang’ (HY). Left: **A** Up-regulated genes and **B** down-regulated genes shared by two materials subjected to Psa treatment (HY-Psa vs. HY-ck and GZ-Psa vs. GZ-ck). Right: Disease-resistance genes of *A. × leiocacarpae* are partly overlapped with **A** the up-regulated genes and **B** the down-regulated genes specific to this material when infected by Psa disease.


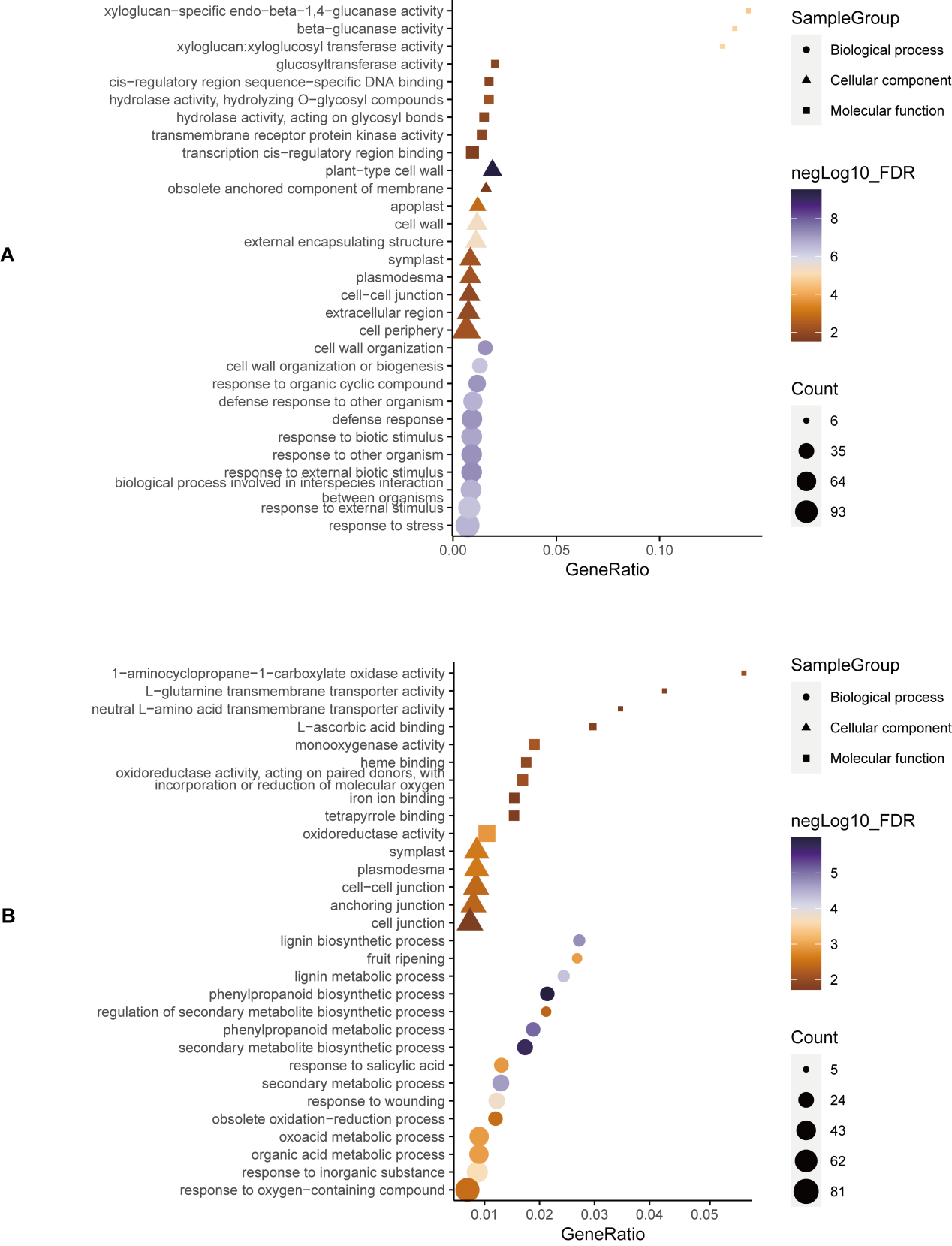


**Supplementary Figure 31.** Scatter plot of enriched GO terms of **A** the specific up-regulated genes and **B** the specific down-regulated genes in *A. × leiocacarpae*. The color and size of the dots represent the range of the FDR and the number of candidate genes mapped to the indicated GO terms, respectively.


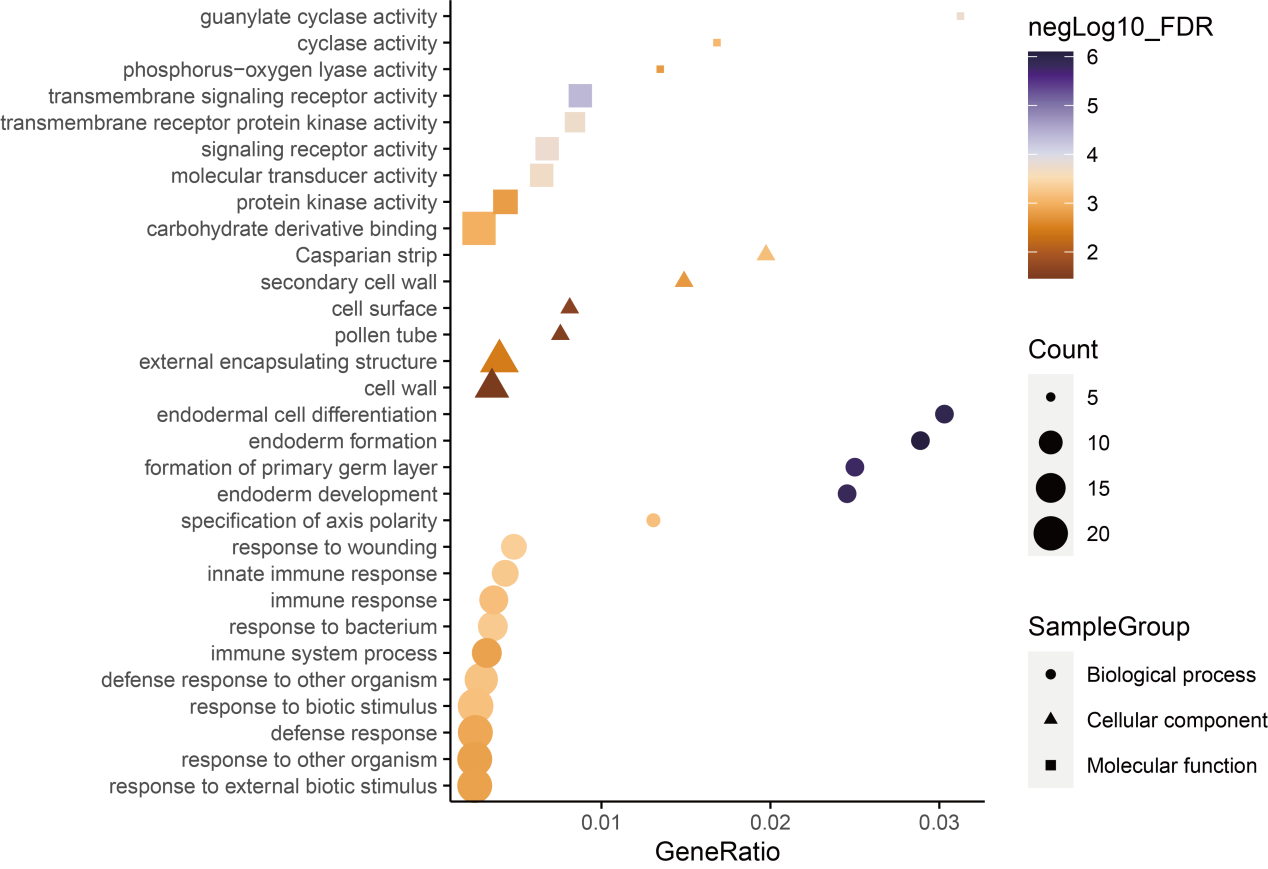


**Supplementary Figure 32.** Scatter plot of enriched GO terms of the materials specific up-regulated genes and (b) the specific down-regulated genes in *A. × leiocacarpae*. The color and size of the dots represent the range of the FDR and the number of candidate genes mapped to the indicated GO terms, respectively.


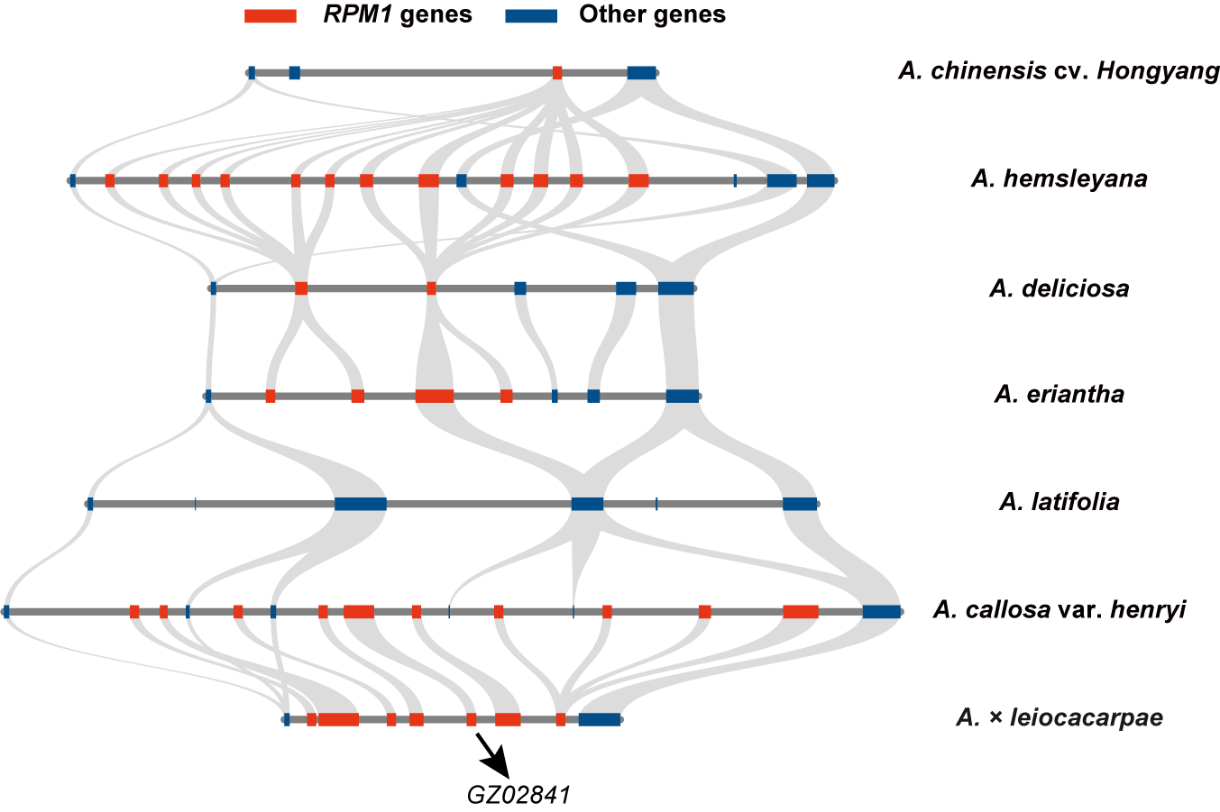


**Supplementary Figure 33.** The landscape of variations of *RPM1* gene clusters on Chr08 in seven *Actinidia* genomes. The *RPM1* genes and other genes are indicated by red and blue rectangles, respectively.

**
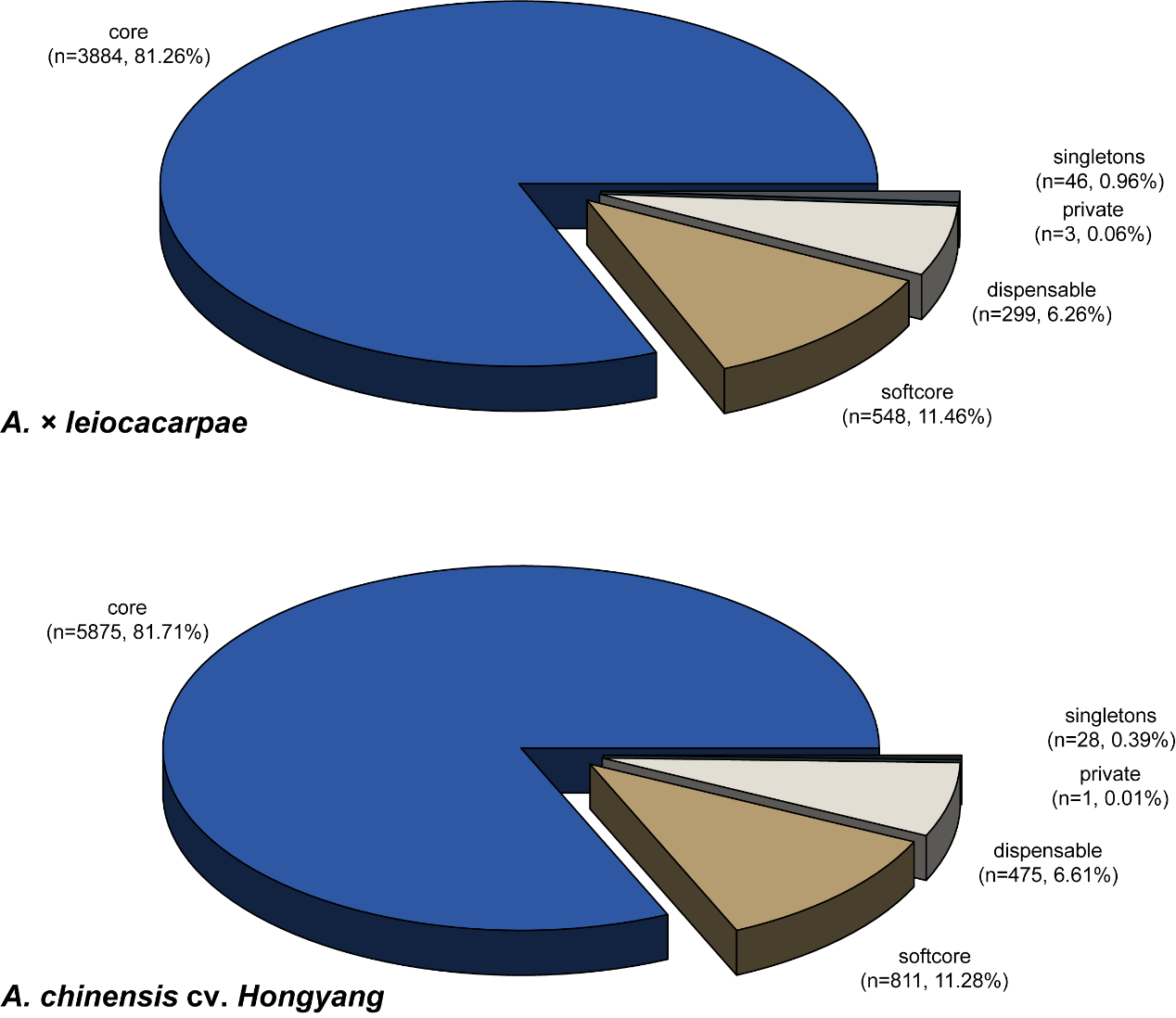
**

**Supplementary Figure 34.** Comparison of different pan-genomic gene family classifications in differentially expressed genes in transcriptomes of *A. × leiocacarpae* and *A. chinensis* cv. ‘Hongyang’.


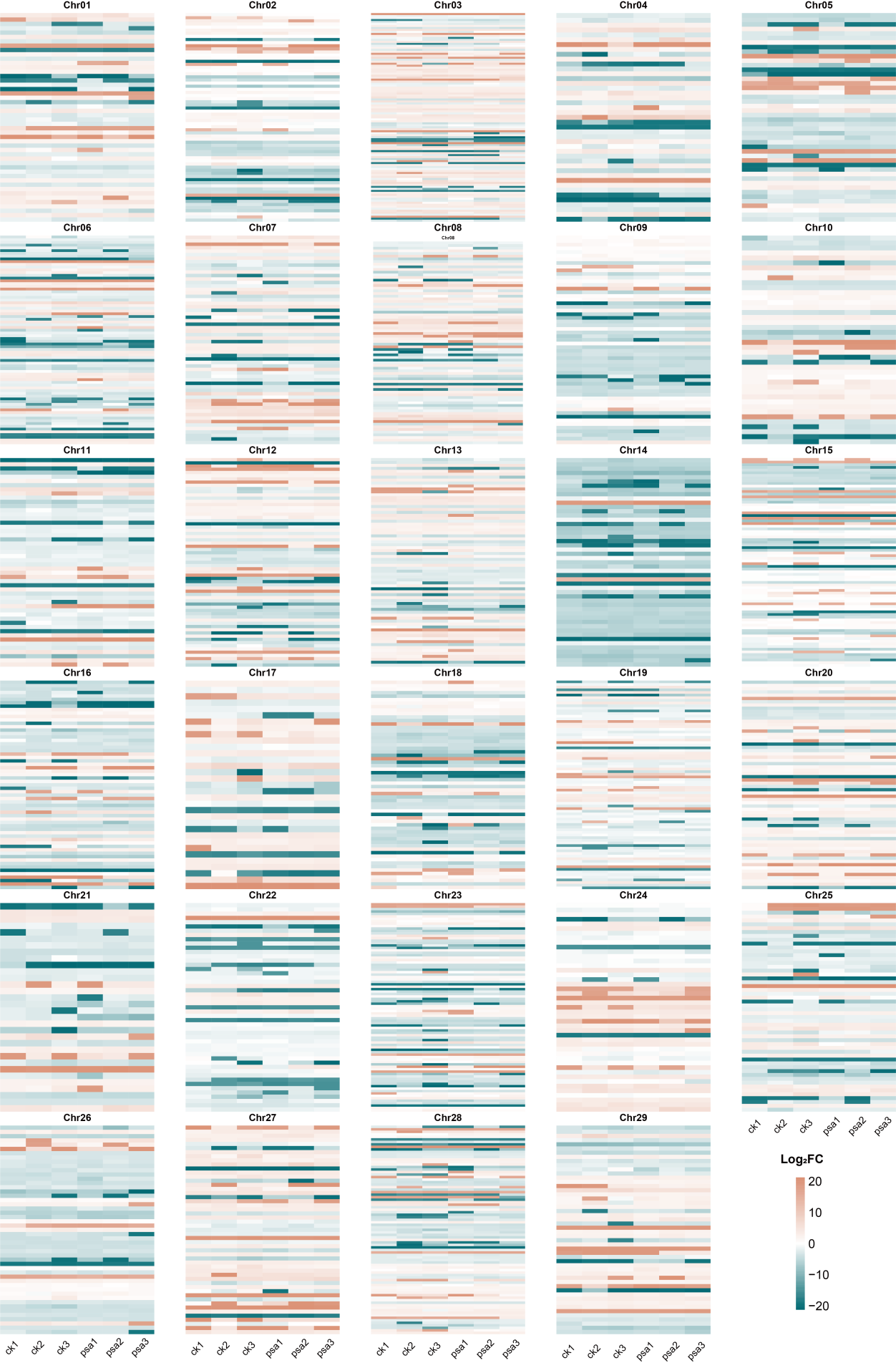


**Supplementary Figure 35.** Expression levels (log2FC) of ASE genes with / without Psa treatment in *A. × leiocacarpae*. The dominance expression pattern of ASEs in GZ1 and GZ2 are colored in agate green and orange, respectively. Psa: Psa; ck: sterile water. “1”, “2” and “3” represent biological duplicates.


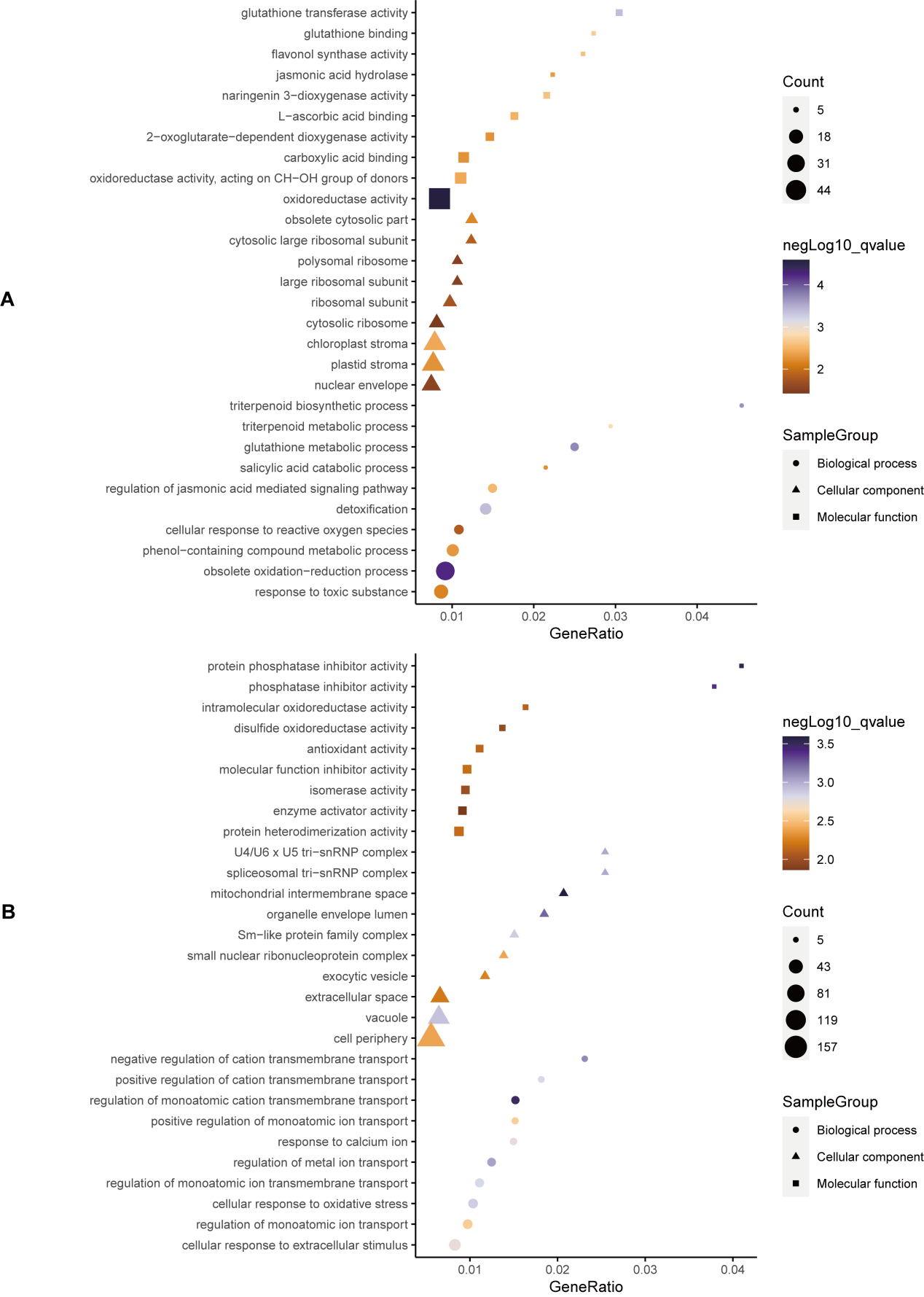


**Supplementary Figure 36.** Scatter plot of enriched Gene ontology (GO) terms of consistent ASE genes in **A** GZ-SG1 and **B** GZ-SG2 genome in A. × leiocacarpae. The color and size of the dots represent the range of the q-value and the number of candidate genes mapped to the indicated GO terms, respectively. The shape of square, triangle and circular represent the three types of GO terms: molecular function, cellular component and biological process, respectively.
